# Broad-Spectrum Inhibitor of Bacterial Polyphosphate Homeostasis Attenuates Virulence Factors and Helps Reveal Novel Physiology of *Klebsiella pneumoniae* and *Acinetobacter baumannii*

**DOI:** 10.3389/fmicb.2021.764733

**Published:** 2021-10-26

**Authors:** Nathan Roberge, Nolan Neville, Katya Douchant, Curtis Noordhof, Nadejda Boev, Calvin Sjaarda, Prameet M. Sheth, Zongchao Jia

**Affiliations:** ^1^Department of Biomedical and Molecular Sciences, Queen’s University, Kingston, ON, Canada; ^2^Department of Pathology and Molecular Medicine, Queen’s University, Kingston, ON, Canada; ^3^Gastrointestinal Disease Research Unit (GIDRU), Department of Medicine, Queen’s University, Kingston, ON, Canada; ^4^Queen’s Genomics Lab at Ongwanada (Q-GLO), Ongwanada Resource Center, Kingston, ON, Canada; ^5^Department of Psychiatry, Queen’s University, Kingston, ON, Canada; ^6^Division of Microbiology, Kingston Health Science Center, Kingston, ON, Canada

**Keywords:** polyphosphate, polyphosphate kinase (PPK), *Acinetobacter baumannii*, *Klebsiella pneumoniae*, virulence factor, anti-virulence therapeutic, gastrointestinal microbiota

## Abstract

Acinetobacter baumannii and *Klebsiella pneumoniae* currently rank amongst the most antibiotic-resistant pathogens, responsible for millions of infections each year. In the wake of this crisis, anti-virulence therapeutics targeting bacterial polyphosphate (polyP) homeostasis have been lauded as an attractive alternative to traditional antibiotics. In this work, we show that the small molecule gallein, a known G-protein βγ subunit modulator, also recently proven to have dual-specificity polyphosphate kinase (PPK) inhibition in *Pseudomonas aeruginosa*, in turn exhibits broad-spectrum PPK inhibition in other priority pathogens. Gallein treatment successfully attenuated virulence factors of *K. pneumoniae* and *A. baumannii* including biofilm formation, surface associated motility, and offered protection against *A. baumannii* challenge in a *Caenorhabditis elegans* model of infection. This was highlighted most importantly in the critically understudied *A. baumannii*, where gallein treatment phenocopied a *ppk1* knockout strain of a previously uncharacterized PPK1. Subsequent analysis revealed a unique instance of two functionally and phenotypically distinct PPK1 isoforms encoded by a single bacterium. Finally, gallein was administered to a defined microbial community comprising over 30 commensal species of the human gut microbiome, demonstrating the non-disruptive properties characteristic of anti-virulence treatments as microbial biodiversity was not adversely influenced. Together, these results emphasize that gallein is a promising avenue for the development of broad-spectrum anti-virulence therapeutics.

## Introduction

Encountering multi-drug resistant bacterial pathogens has become a common occurrence in modern medicine, and the frequency of identification is on the rise. As antibiotic development has stagnated over the past several decades (Ventola, 2015; Andrei et al., 2019), many species have developed effective resistance strategies to circumvent conventional therapeutics (Rossolini et al., 2014; Dickey et al., 2017). To address this mounting threat, novel classes of drugs targeting an organism’s ability to infect, disseminate, and persist within a host, termed virulence factors, have been proposed as an alternative to antibiotics (Cross, 2008). This approach aims to attenuate virulence but avoid bactericidal activity, thereby reducing the selective pressures which are an inevitable consequence of traditional antibiotic use (Cegelski et al., 2008; Totsika, 2017). Furthermore, many antibiotics are not pathogen specific and will thus devastate commensal microbial communities. This can lead to re-infection, or increased susceptibility to other infectious agents including *Clostridiodes difficile* (Keeney et al., 2014), yet anti-virulence therapeutics should avoid these disastrous consequences (Totsika, 2016).

It is for these reasons, that polyphosphate (polyP) research has re-emerged at the forefront of the anti-virulence discussion, as to exploit a novel strategy to combat antibiotic resistant pathogens. PolyP is a ubiquitous biopolymer consisting of linearly arranged inorganic phosphate residues connected through phosphoanhydride bonds (Brown and Kornberg, 2008). The enzymes of the polyphosphate kinase (PPK) family are responsible for overseeing cellular polyP homeostasis (Zhu et al., 2005), with polyphosphate kinase 1 (PPK1) serving as the principle regulator of biosynthesis, along with polyphosphate kinase 2 (PPK2) (Zhang et al., 2002). In addition to being highly conserved across Gram positive and Gram negative bacterial species (Rashid et al., 2000; Zhang et al., 2002), in many pathogenic bacteria, polyP modulation and upstream PPK regulatory networks have been intimately linked with virulence factor mechanisms and antibiotic susceptibility (Fraley et al., 2007; Gangaiah et al., 2010; Chuang et al., 2013; Batten et al., 2016; Dahl et al., 2017; Neville et al., 2021). As the enzymes of the PPK family have no known homolog in higher eukaryotes (Gómez-García and Kornberg, 2004), it is unsurprising that they have been lauded as attractive targets for the design of anti-virulence therapeutics. We recently demonstrated the feasibility of PPK drug targeting in the species *Pseudomonas aeruginosa*, where the small molecule gallein, was shown to act as a dual-specificity inhibitor of PPK1 and all three PPK2 isoforms in this bacterium (Baijal and Downey, 2021; Neville et al., 2021). The resulting attenuation of virulence factors including swimming motility, toxin secretion, and biofilm formation culminated in protection against bacterial challenge in a *Caenorhabditis elegans* model of infection (Neville et al., 2021). These findings cemented gallein as the first validated inhibitor of its kind capable of both PPK1 and PPK2 inhibition in a pathogenic species. It remains to be determined, whether gallein is specific for *P. aeruginosa* or if it exhibits broad-spectrum against other bacterial pathogens.

The Gram-negative opportunistic pathogens *Acinetobacter baumannii* and *Klebsiella pneumoniae* rank amongst the most antibiotic-resistant bacteria, each responsible for millions of infections each year (World Health Organization, 2017). This public health crisis led the World Health Organization (WHO) to include these species in its first list of priority pathogens in urgent need of novel antimicrobials to manage infections (World Health Organization, 2017). While the myriad of cellular responses shown to require intracellular polyP biosynthesis have been well characterized in *Escherichia coli* and *P. aeruginosa* (Shiba et al., 1997; Rashid et al., 2000; Fraley et al., 2007; Dahl et al., 2017), details surrounding the contributions of the polyP pathways are conspicuously lacking for *A. baumannii* and *K. pneumoniae*. Both these bacteria rely on several common virulence factors (Russo et al., 2011; Tipton et al., 2015; Pakharukova et al., 2018; Zheng et al., 2018), yet little is known about the physiological involvement of the PPK enzymes. *K. pneumoniae* encodes a PPK1 (Supplementary Figure 1) and class 1 PPK2^1^ (Supplementary Figure 2). *A. baumannii* however presents with a rare instance of encoding two individual PPK1 isoforms, PPK1A and PPK1B (Supplementary Figure 1), and a class 2 PPK2^2^ (Supplementary Figure 2). While PPK1 enzymes from *Acinetobacter* sp. have indeed been characterized, these studies focused on the PPK1B or PPK2 isoforms (Gavigan et al., 1999; Trelstad et al., 1999; Itoh and Shiba, 2004; Gautam et al., 2021), leaving the functional properties of PPK1A largely unknown. Previous studies have also noted a phenotypically distinct phase-variation influencing virulence factors in *A. baumannii* (Tipton et al., 2015; Tipton and Rather, 2017; Ahmad et al., 2019), though whether discrepancies in PPK1 intracellular function or regulation exist within subpopulations is unknown. Here we assess the newly identified dual-specificity PPK inhibitor gallein for broad-spectrum virulence factor inhibition, and in turn employ it as a tool to study bacterial physiology in *A. baumannii* and *K. pneumoniae*.

## Materials and Methods

### Strains and Growth Conditions

All strains, primers, and plasmids used in this study are listed in Supplementary Table 1. *K. pneumoniae* strain KPNIH1 and *A. baumannii* strain AB5075 and its various PPK1 transposon knockouts were obtained from the Manoil lab three-allele library (University of Washington). *K. pneumoniae* was cultured aerobically at 37°C in lysogeny broth (LB—Miller; Bioshop). Unless otherwise specified, *A. baumannii* was cultured aerobically in LB containing no additional NaCl (10 g/L tryptone, 5 g/L yeast extract). For all phenotype assessments, *K. pneumoniae* and *A. baumannii* were passaged at least 2–3 times prior to each assay, and plates or cultures were no longer used after 5 days.

### *A. baumannii* Phase-Variant Selection and Growth Conditions

*A. baumannii* phase-variant colonies were grown on solid media plates containing 10 g/L tryptone, 5 g/L of yeast extract, and 0.8% Agar (Tipton et al., 2015). Phase-variants were confirmed using a light microscope viewed under oblique lighting conditions. Single colonies of opaque or translucent cells were cultured overnight at 37°C with shaking at 150 rpm. When applicable, phase segregation was confirmed through CFU/mL analysis (Tipton et al., 2015).

### Cloning, Heterologous Expression, and Purification of Proteins

Codon optimized *K. pneumoniae* KPNIH1 PPK1 and *A. baumannii* AB5075 PPK1B were obtained from Integrated DNA Technologies (IDT), and Thermo GeneArt, respectively. *E. coli* PPK1 was provided in a pUC57 storage vector by Dr. F. Chavez (Universidad de Chile). *A. baumannii* PPK1A, and *S. marcescens* PPK1 were cloned off genomic templates of AB5075 and ATCC 31453, respectively. All genes were subcloned into pET28a with a C-terminal His_6_-tag for inducible expression, with the exception of PPK1B, which was cloned into HT7, a pET16b-derived expression vector that yields an N-terminal His_6_-tag. Given primer sequence similarity between *E. coli*, *S. marcescens*, and *K. pneumoniae* PPK1s, successful transformants were identified using T7 promoter specific primers T7 chk_fwd and rev. All primer sequences and restriction enzymes used are listed in Supplementary Table 1. All enzyme expression was induced as previously described (Neville et al., 2021). All PPK1s were purified in the same manner as previously described (Zhu et al., 2005; Neville et al., 2021) with some minor deviations. In brief, PPK1 expressing cultures of BL21 were harvested by centrifugation and resuspended in Buffer A (50 mM Tris-HCl adjusting pH as appropriate, 10% sucrose, 5 mM 2-mercaptoethanol, and 20 mg lysozyme). All buffers used were adjusted to a pH of 7.5, with the exception of PPK1A and PPK1B purification in which pH 8.0 and 8.5 were used, respectively. Cells were incubated on ice for 45 min, and then heat shocked at 37°C for 10 min. Supernatant was removed by centrifugation (18k, 30 min). The pellet was then resuspended in Buffer B (50 mM Tris-HCl, 10% sucrose, 5 mM MgCl_2_, 5 mM 2-mercaptoethanol, and 10 μg/mL DNase1/RNaseA) and resuspended using a sonicator bath. To extract PPK1 from the membrane fraction, 1/10 volume of 1 M Na_2_CO_3_ and 1 M final concentration of solid KCl were added to the lysate, and the mixture was stirred for 1 h at 4°C. Cell debris was removed by centrifugation (18k, 30 min) and the supernatant was diluted with an equal volume of Milli-Q water. The supernatant was then applied to a Ni^2+^-NTA column pre-equilibrated with Buffer C (50 mM Tris-HCl, 500 mM NaCl, and 3 mM 2-mercaptoethanol), and allowed to mix with the resin for 30–40 min at 4°C. Protein was eluted from the column with an imidazole gradient dissolved in Buffer C (10, 50 × 2, 75, 100, 200, and 300 mM imidazole concentration fractions). Fractions containing PPK1 were determined through sodium dodecyl sulfate polyacrylamide gel electrophoresis (SDS-PAGE). Protein containing fractions were pooled and dialyzed overnight at 4°C against Buffer D (20 mM Tris-HCl, 0.15 M NaCl, 15% glycerol, and 5 mM DTT). The exception was PPK1A, in which the buffer contained 5% glycerol. Protein was then aliquoted, flash frozen, and stored in liquid nitrogen.

*A. baumannii* and *K. pneumoniae* PPK2 were cloned into HT29, a pET16b expression vector derivative with N-terminal maltose binding protein (MBP) and His_6_ fusion tags (Supplementary Table 1). All primers and restriction enzymes used are again listed in Supplementary Table 1. PPK2 enzymes were purified as previously described (Neville et al., 2021), with the exception of *K. pneumoniae* PPK2, in which the MBP fusion was left intact.

### IC_50_ and Kinetic Assays

All IC_50_ assays contained final equimolar amounts (50 nM) of PPK to ensure valid comparison. PolyP synthesis activity was assayed as previously described (Neville et al., 2021). PPK reaction buffer was prepared containing 50 mM HEPES-NaOH pH 7.5, 40 mM (NH_4_)_2_SO_4_, 4 mM MgSO_4_, 60 mM creatine phosphate, 0.0062 mg/mL creatine kinase (Sigma, rabbit muscle), and 5 mM ATP. Reactions were carried out in 100 μL volumes, supplemented with the appropriate volume of 10 mM gallein stock dissolved in DMSO, incubated at 37°C for 30 min, after which 50–90 μL aliquots of reaction mixture were added to 1 mL of 6 mg/L toluidine blue dye dissolved in 40 mM acetic acid. Reactions were also carried out in the absence of creatine phosphate and creatine kinase 2 h at 37°C to counter screen for off-target creatine kinase inhibition (Supplementary Figure 3). A_630__/__530_ ratio was then recorded, and the amount of polyP synthesized was calculated using a standard curve (Neville et al., 2021). For kinetic experiments, reactions were carried out in the same way with the exception of varying ATP concentration as indicated, as well as reducing reaction time were appropriate (Supplementary Figure 3). For the *A. baumannii* PPK1s, placement of the His_6_-tag was found to influence polyP biosynthetic activity. N-terminally His_6_-tagged PPK1B showed greater activity than C-terminally tagged protein (Supplementary Figure 4). Thus, N-His_6_-PPK1B was chosen for comparison. No polyP biosynthetic activity was detected for N-terminally His_6_-tagged PPK1A recombinantly expressed in the HT7 vector backbone (data not shown). This finding is consistent with *E. coli* N-terminally His_6_-tagged PPK1 also being catalytically inactive (Zhu et al., 2003). Curves were fit using GraphPad Prism version 9.

Nucleotide phosphorylation assays of PPK2 were carried out as previously described (Neville et al., 2021). In brief, reaction buffer was prepared containing 50 mM Tris-HCl pH 7.5, 10 mM (NH4)2SO4, 10 mM MgCl2, 5 mM polyP45 (Sigma; in terms of individual Pi monomers), and nucleotide (ADP or AMP) at 5 mM for IC50 reactions or as indicated for kinetic experiments. 100 μL reactions were incubated at 37°C for 30 min, then 5 μL aliquots were withdrawn and added to 1,000 μL of 6 mg/L toluidine blue dissolved in 40 mM acetic acid to quench the reaction and quantify the polyP that remained. The A630/530 ratio was then recorded, and the values obtained were corrected by subtracting the corresponding blanks (all ingredients except enzyme). The amount of polyP consumed was calculated by subtracting the amount present after reaction from that present in the initial reaction mix, as calculated via a standard curve. Each monomer of Pi consumed from polyP equates to one nucleoside phosphorylated. Curves were fit using GraphPad Prism version 9.

### Intracellular Polyphosphate Accumulation Assay

*K. pneumoniae* polyP levels were quantified as previously described (Dahl et al., 2017; Neville et al., 2021) with slight modifications. In brief, 5 mL LB media cultures of *K. pneumoniae* were grown at 37°C overnight in the presence of 100 μM of gallein or equivalent volume of DMSO. Low-Pi MOPS minimal media incubation was omitted as this procedure was found to reduce intracellular polyP derived signal in *K. pneumoniae*. Cultures were centrifuged for 10 min at 3,900 rpm to pellet cells. Cells sufficient to yield 200 μg of total cellular protein were resuspended in GITC lysis buffer and boiled for 10 min at 95°C. PolyP was purified and converted to ATP via silica spin columns and PPK digestion as described (Dahl et al., 2017), with the exception of using 50 nM *P. aeruginosa* PPK2A as opposed to *E. coli* PPK1 for ATP conversion (Neville et al., 2021). An Invitrogen ATP determination kit (Thermo Fisher Scientific) was used per the manufacturer’s instructions for ATP quantification. Luminescence was recorded on a SpectraMax iD3 microplate reader (Molecular Devices). PolyP extraction and quantification of chemostat samples were carried out in an identical manner using 1 mL aliquots of culture. *A. baumannii* polyP was extracted following a similar procedure with the following exceptions. *A. baumannii* 5 mL LB or no salt LB media where applicable cultures were grown at 37°C overnight. Cultures were then resuspended in low-Pi MOPS minimal medium as described (Dahl et al., 2017; Neville et al., 2021) with 100 μM gallein or an equivalent volume of DMSO. PolyP extraction and quantification were then carried out as described above, using cells sufficient to yield 80 μg of total cellular protein.

### Polyphosphate Electrophoresis

PolyP samples were purified as described above. The purified eluate was mixed with loading dye (10 mM Tris-HCl pH 7, 1 mM EDTA, 30% glycerol, bromophenol blue) and electrophoresed on 15.8% TBE-urea gels as described (Bondy-Chorney et al., 2020; Neville et al., 2021). The volume loaded for each sample was normalized with respect to total protein content of the original lysate as determined with the Bradford assay. PolyP was visualized via negative DAPI staining.

### Growth Kinetic Assay

Cultures of *K. pneumoniae* or *A. baumannii* were grown to mid-log phase. Resulting cultures were diluted to an OD_600_ of 0.3, of which 1 μL was then used to inoculate 100 μL of LB or no salt LB where applicable, supplemented with 100 μM Gallein or an equivalent volume of DMSO in a flat bottom 96 well TC treated plate. Cultures were grown aerobically at 37°C with shaking for 20 h. Readings were recorded every 30 min on a SpectraMax iD3 microplate reader (Molecular Devices).

### Biofilm Assay

To assess biofilm growth, 500 μL of LB or no salt LB media was added to 5 mL polypropylene culture tubes, which were then inoculated with a single colony of *K. pneumoniae* or *A. baumannii* AV-T cells, respectively. Cultures were incubated at 37°C overnight with shaking in the presence of an indicated concentration of gallein, 100 μg/mL ampicillin, or an equivalent volume of DMSO. Cultures were then removed from the tubes, diluted 1:10 and growth was quantified via OD_600_. The resulting culture tubes were next washed with 1 mL of distilled water. Water was decanted and 800 μL of 0.1% crystal violet dye was added for 10–15 min to stain the remaining biological material. The dye was removed, washed once more with 1 mL of distilled water, solubilized in 800 μL of 30% acetic acid to record absorbance at 570 nm. Biofilm readings were normalized with respect to bacterial culture growth where applicable.

### RNA Isolation

Cultures of *A. baumannii* strains were grown in no salt LB 37°C with shaking to an OD_600_ of ∼0.5–0.7. 500 μL of culture was harvested and the remaining culture was resuspended in low-Pi MOPS minimal medium for another 2 h at 37°C. The cells were harvested by centrifugation, and RNA from both time points was isolated using a Qiagen RNeasy Mini kit with optional on column DNase digestion as per the manufacturer’s instructions (Qiagen). RNA concentration and purity were assessed using a Molecular Devices quick drop spectrophotometer.

### Quantitative Real-Time PCR

Total RNA (900 ng) purified from *A. baumannii* was incubated for 5 min at 42°C with gDNA Wipeout Buffer (Qiagen). The resulting RNA was converted into cDNA using the QuantiTect Reverse Transcription kit with random primers and Quantiscript Reverse Transcriptase. Samples were incubated for 30 min at 42°C as per the manufacturer’s instructions (Qiagen). cDNA reactions were then diluted 1:10 with sterile water. Oligonucleotide primer pairs for quantitative real-time PCR (qRT-PCR) were generated using the Primer-BLAST server.^3^ Primers were designed to amplify ∼100–200 bp fragments for each gene. qRT-PCR was performed using *Power*SYBR Green PCR Master Mix (Thermo Fisher Scientific) with a Biorad CFX-96 cycler. The following cycling parameters were used to amplify and quantify the fragments: 95°C for 10 min, followed by 40 cycles of 95°C for 15 s, and 60°C for 60 s. Melting-curve data were collected to ensure proper amplification of target genes. Data were generated from three separate RNA isolations, cDNA preparations, and from technical triplicates for each primer set. The relative expression of each gene was determined by comparing target gene expression with reference gene *clpX* as previously described (Tipton and Rather, 2017).

### *A. baumannii* Surface Associated Motility Assay

Surface associated motility was conducted as previously described (Tipton et al., 2015) with some modifications. Briefly, motility plates were prepared fresh the day of the assay by autoclaving 10 g/L tryptone, 5 g/L yeast extract, 5 g/L NaCl, and 0.3% agarose. Each plate was made using 20 mL of the medium and were supplemented with 100 μM gallein or an equivalent volume of DMSO where indicated. Prior to use, the motility plates were dried for 25–30 min in a biological safety cabinet to ensure no surface moisture present. The center of each plate was then inoculated with a single colony of *A. baumannii* VIR-O cells. The plates were then placed in an air-tight container and incubated at 37°C overnight and cellular translocation diameter was imaged and measured electronically.

### *C. elegans* Fertility Assay

Wild-type N2 *C. elegans* were obtained from the Caenorhabditis Genetics Center. Virulence of the *A*. *baumannii* strains was assessed using *C*. *elegans* fertility as previously described with some modifications (Vallejo et al., 2015; Ahmad et al., 2019). Briefly, the *C*. *elegans* were cultivated on Nematode Growth Medium (NGM) plates seeded with a lawn of *E. coli* OP50. Physiologically synchronized worms were grown at 25°C to L4 larval stage. The fertility assay was performed by inoculating a single L4 stage worm per NGM plate seeded with 6.7–7.0 × 10^6^ colony forming units of the appropriate strain and containing 100 μM gallein or equivalent volume of DMSO. Plates were incubated at 25°C for 24 h at which point the adult worm was removed and transferred to another plate of the same bacterial strain and seeded the same way. Nematode progeny was counted daily for 2 days, and 48 h following the removal of the adult worm. Seven independent replicates were performed for each strain of *A. baumannii*, and three for OP50 negative controls.

### Growth and Treatment of Bacterial Cultures in Chemostat Model

33 different bacterial strains were cultured according to established methods and inoculated a twin-vessel chemostat model (Martz et al., 2017). The twin-vessel chemostat system allows for simultaneous operation of two vessels inoculated with the same bacterial cultures (Guzman-Rodriguez et al., 2018). Communities were allowed to colonize for 10 days (growth phase) before perturbations. Beginning on day 10, one chemostat was injected with gallein and the other with the DMSO vehicle for a period of 10 days (treatment phase). Following the treatment phase, both chemostats were allowed another 8 days of growth (washout phase). Samples were collected daily from each chemostat throughout all three phases, including biofilm waste outflow tubing, and stored at −80°C until analysis.

### 16S Microbiome Sequencing

An aliquot (200 μL) of the sample was centrifuged at 13,000 *g* to pellet the bacterial cells prior to DNA extraction using E.Z.N.A. Stool DNA Kit (Omega Bio-Tek). Using 5 ηg of DNA as input, 16S hypervariable regions were amplified using both primer sets (V2-4-8 and V3-6, 7–9) in the Ion 16S Metagenomics Kit (Thermo Fisher Scientific) and 18 PCR cycles on an Applied Biosystems GeneAmp PCR System 9700. PCR products were ligated with Ion Xpress Barcode (Thermo Fisher Scientific) to allow for multiplexing, and libraries were quantified using the Ion Universal Library Quantitation Kit (Thermo Fisher Scientific) on a Viia7 Real-Time PCR machine. Templating (40 pM) and chip loading were performed on the Ion Chef system using the Ion 510 and Ion 520 and Ion 530 Kit-Chef. Samples were multiplexed on an Ion 530 chip and sequenced using the Ion GeneStudio S5 Plus Semiconductor Sequencer. The raw data of all 16S libraries generated during this study is publicly available at the Sequence Read Archive (SRA) portal of NCBI under accession number PRJNA758409.

### Chemostat Data Processing and Statistics

Samples were preprocessed using the dada2 v1.19.2 pipeline^4^ (Callahan et al., 2017; Callahan et al., 2019). Assessment of read quality facilitated read length truncation (truncLen = 150), to allow for varying lengths of variable regions, read trimming (trimLeft = 15), as recommended for Ion Torrent data, and removal of chimeras (method = consensus), which relies on a consensus for each variant. Reads for the chemostat vessels and waste outflow tubing were rarefied at 18,470 and 44,430, respectively. Rarefaction was validated by calculating the coefficient of variation in diversity metrics over 10 iterations, where day 15 and 25 did not meet the threshold and were removed. Taxonomic annotation was conducted using RPD18 using dada2’s Naïve Bayesian classifier (Callahan, 2020) where the classifier allowed reverse complement matching.

We calculated alpha and beta diversity, quantified by Shannon index and Bray-Curtis, respectively, using Phyloseq v1.34.0 (McMurdie and Holmes, 2013) and vegan v2.5-7 (Oksanen et al., 2020) packages in R v4.0.2 (R Core Team, 2020). Relative alpha diversity was calculated using the mean alpha diversity for the corresponding chemostat across the duration of the experiment. To investigate the fixed effects and interactions of treatment and time on alpha diversity, a linear mixed model using the lme4 v1.1-27 package (Bates et al., 2015) including the random effects of the chemostat and model selection by ANOVA. Principal coordinate analysis was used for ordination, where the comparison of centroids and distribution was conducted with PERMOVA using 99 permutations and the ellipses drawn at a 95% confidence level.

## Results

### Gallein Inhibits *Enterobacteriaceae* PPK1s

**FIGURE 1:**
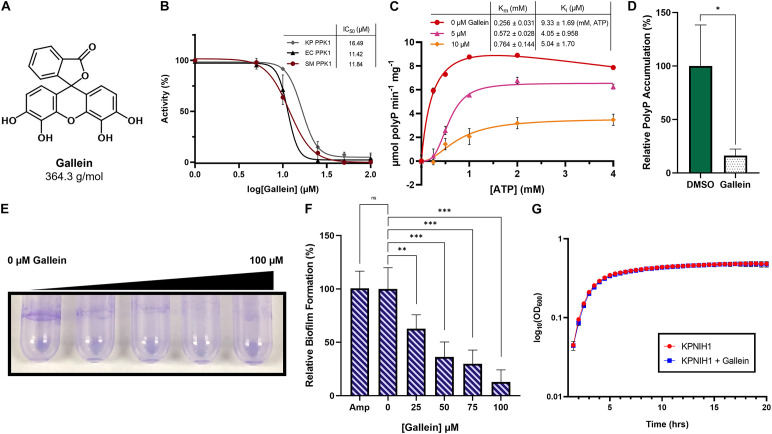
Gallein inhibits *Enterobacteriaceae* PPK1s *in vitro*, and reduces intracellular polyP accumulation, biofilm formation in *K. pneumoniae*. **(A)** Chemical structure of gallein. **(B)** Dose-dependent inhibition of polyP synthesis by purified PPK1 enzymes from *Klebsiella pneumoniae* (KP), *Escherichia coli* (EC), and *Serratia marcescens* (SM). **(C)** Enzyme kinetic analysis of *K. pneumoniae* PPK1. Units are reported in specific activity (μmol per min per mg PPK). Uninhibited curve fit using substrate inhibition model (V^o^ = Vmax*[S]/(K_m_ + [S]*(1 + [S]/Ki)) where S is substrate), while inhibited curves fit using sigmoidal model (V^o^ = Vmax*[S]^h/(K_m_^ h + [S]^h), where S is substrate, and h is the Hill coefficient). **(D)** Effect of 100 μM gallein treatment on starvation-induced intracellular polyP accumulation in *K. pneumoniae*. **(E)** Representative image of gallein treatment on *K. pneumoniae* biofilm formation. Image is representative of *n* = 3 assays. **(F)** Dose-dependent effects of gallein and 100 μg/mL ampicillin on *K. pneumoniae* biofilm formation. **(G)** Growth kinetics in lysogeny broth of *K. pneumoniae* KPNIH1 in the presence or absence of gallein. Symbols are as follows: ns, *p* > 0.05; ^∗^*p* < 0.05; ^∗∗^*p* < 0.01; ^∗∗∗^*p* < 0.001. For **(D)**, two-way Unpaired *t*-test, *n* = 3; for **(F)**, one-way ANOVA, Tukey’s multiple comparison test, *n* = 6, Amp *n* = 3.

Given that the small molecule gallein (Figure 1A) is a known polyP biosynthesis inhibitor in *P. aeruginosa* (Neville et al., 2021), we first attempted to identify whether gallein treatment could inhibit PPK1s from other priority species. Beginning with the *Enterobacteriaceae* family of bacteria, PPK1s from *K. pneumoniae*, *E. coli*, and *Serratia marcescens* were recombinantly expressed, purified, and screened for gallein inhibition. Gallein treatment inhibited PPK1 catalyzed polyP synthesis from all three species in a dose-dependent manner (Figure 1B). Gallein treatment also demonstrated dose-dependent inhibitory action against *K. pneumoniae* PPK2 ATP synthesis (Supplementary Figure 2), reminiscent of the dual-specificity PPK inhibition observed in *P. aeruginosa*. Enzymatic inhibition was further illustrated with the *K. pneumoniae* PPK1 isoform, where the addition of low micromolar concentrations of gallein increased the Michaelis constant (K_m_) for ATP and reduced the K_i_ for ATP (Figure 1C).

### Gallein Reduces Intracellular PolyP and Attenuates Virulence Factors of *K. pneumoniae*

Following the successful inhibition of *K. pneumoniae* PPK1 *in vitro*, we then pivoted toward the inhibition of intracellular polyP biosynthesis. As expected, gallein treatment significantly reduced intracellular polyP accumulation in *K. pneumoniae* KPNIH1 (Figure 1D). This was accompanied by a significant attenuation of biofilm formation, in which an increasing concentration gradient of gallein reduced exopolymer staining in a dose-dependent manner (Figures 1E,F). In contrast, biofilm formation was not reduced upon treatment with 100 μg/mL of the antibiotic ampicillin (Figure 1F). Importantly, despite metabolically influencing the cells to reduce biofilm formation, gallein treatment did not reduce cellular viability as determined through growth assay (Figure 1G and Supplementary Table 2).

### Gallein Inhibits PPK1s and Reduces Intracellular PolyP Accumulation in *A. baumannii*

**FIGURE 2:**
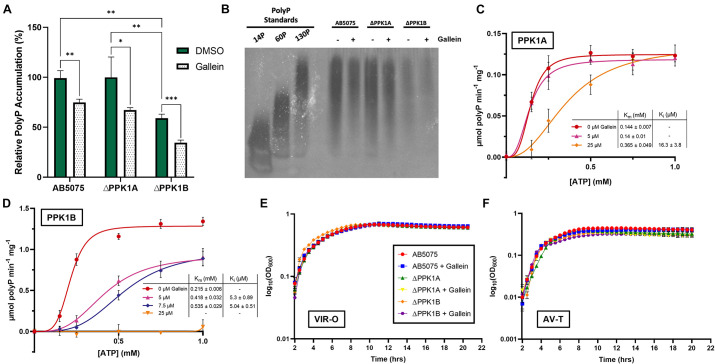
Gallein inhibits *A. baumannii* PPK1s *in vitro* and intracellularly but does not attenuate growth kinetics **(A)** Effect of gallein treatment on starvation-induced intracellular polyP accumulation **(B)** Bacterial polyP extracts treated with 100 μM gallein or DMSO vehicle analyzed via electrophoresis on Tris-borate-EDTA (TBE)-urea gel following negative DAPI staining. Image is representative of *n* = 3 gels. **(C,D)** Enzyme kinetic analysis of PPK1A **(C)** and PPK1B **(D)**. Units are reported in specific activity (μmol per min per mg PPK). Curves fit using sigmoidal model (V^o^ = Vmax*[S]^h/(K_m_^ h + [S]^h), where S is substrate, and h is the Hill coefficient). **(E,F)** Growth kinetics in lysogeny broth of VIR-O **(E)** and AV-T **(F)** phase-variants in the presence or absence of gallein. Symbols are as follows: *p* > 0.05; ^∗^*p* < 0.05; ^∗∗^*p* < 0.01; ^∗∗∗^*p* < 0.001 (two-way ANOVA, Tukey’s multiple-comparison test, *n* = 3). All data points are the average from triplicates; error bars are ± SD.

*A. baumannii* is currently the number one critical priority multi-drug resistant pathogen as outlined by the WHO (World Health Organization, 2017). We therefore chose to direct our focus toward this species, to ascertain whether gallein would be a viable alternative in combatting this threat. We initially determined that *A. baumannii* does indeed encode both a PPK1 and a class 2 PPK2 enzyme (see text footnote 2). To our surprise however, closer inspection of this organism’s genome revealed that it encodes a second putative PPK1 isoform. This gene was dubbed *ppk1A* (ABUW_2072) encoding PPK1A, while the previously characterized known isoform was named *ppk1B* (ABUW_2907) encoding PPK1B. The protein product homologs comprised ∼67% sequence identity (Supplementary Figure 1). This presented a rare instance of two PPK1 enzymes encoded by a single bacterium, a situation which has been identified only once prior in the environmental bacterium *Ralstonia eutropha* (Tumlirsch et al., 2015). To characterize any functional or physiological discrepancies between the two enzymes, we obtained transposon derived null-allele strains of AB5075 parent from the University of Washington three-allele library.^5^ Correct transposon insertion of ABUW_2072-167:T26 (ΔPPK1A) and ABUW_2907-134:T26 (ΔPPK1B) were confirmed via PCR as per supplier instruction (Supplementary Figure 5). We show that intracellular polyP accumulation was influenced both by single PPK1 knockout as well as gallein treatment. The ΔPPK1A strain did not display any significant reduction in polyP accumulation with respect to the parent, yet the ΔPPK1B strain, however, was attenuated in polyP accumulation (Figure 2A). Notably, cumulative polyP reduction was observed in all three strains upon gallein treatment, indicating both PPK1 enzymes are responsible for polyP biosynthesis. These trends were further visualized through polyP gel electrophoresis (Figure 2B). Next, we recombinantly expressed and purified PPK1A and PPK1B for enzymatic activity analysis. Both PPK1A and PPK1B were capable of *in vitro* polyP synthesis, and also exhibited inhibition by gallein (Figures 2C,D). Gallein treatment also demonstrated dose-dependent inhibition against *A. baumannii* PPK2 ADP synthesis (Supplementary Figure 2). While substrate inhibition is a known phenomenon inherent to the PPK family (Dahl et al., 2017; Neville et al., 2021), in the case of PPK1A and PPK1B, the influence of this effect was rather potent (Supplementary Figure 3). This restricted the useable substrate concentration range for kinetic analysis to 1 mM ATP. Under these conditions, PPK1A maximal specific activity was approximately 10-fold lower than PPK1B. This decrease in activity is nonetheless consistent with the observation that the ΔPPK1B strain accumulates significantly less intracellular polyP (Figures 2A,B). Both enzymes have similar K_m_ values for ATP (Figures 2C,D), and the observed fold change in activity was not due to differing PPK1A nucleotide specificity (Supplementary Figure 3).

### *A. baumannii* PolyP Accumulation and PPK1 Transcription Display Phase-Variant Specific Regulation

**FIGURE 3:**
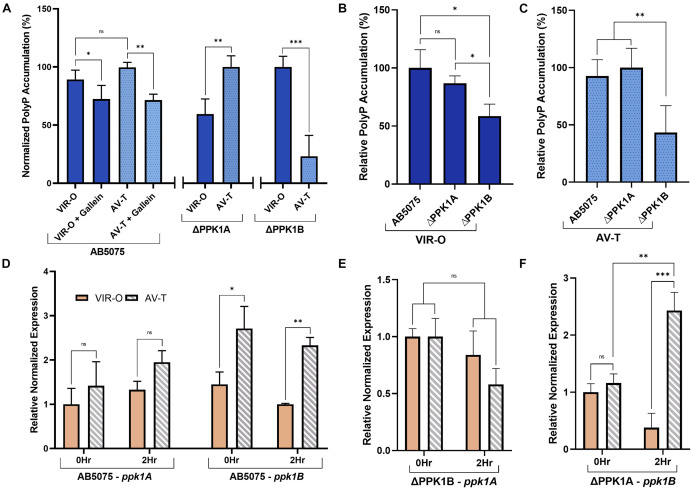
*A. baumannii* polyP accumulation and PPK1 transcription display phase-variant specific regulation. **(A)** Relative phase-variant specific starvation-induced polyP accumulation of all strains. Parent and individual PPK1 knockout strains polyP levels are normalized with respect to the higher signal phase-variant. **(B,C)** Starvation-induced polyP accumulation of VIR-O **(B)** and AV-T **(C)** phase-variants between parent and PPK1 knockout strains. Data points are the average from triplicates or more; error bars are ± SD. **(D)** Phase-variant specific relative normalized expression of *ppk1A* and *ppk1B* in *A. baumannii* parent strain following 2 h phosphate-starvation. All AV-T as well as VIR-O *ppk1* transcription is not significantly different. **(E,F)** Phase-variant specific relative normalized expression of *ppk1A* from PPK1B and *ppk1B* from PPK1A respectively. **(D–F)** Are normalized with respect to *clpX*. Data points are the averages from 3 independent RNA isolations, error bars are ± SEM. Symbols are as follows: ns, *p* > 0.05; ^∗^*p* < 0.05; ^∗∗^*p* < 0.01; ^∗∗∗^*p* < 0.001. For **(A)**, two-way ANOVA, conTukey’s multiple-comparison test, *n* = 3, VIR-O/VIR-O + Gallein *n* = 5; **(B,C)**, two-way Unpaired *t*-test, *n* = 3; **(D–F)**, two-way Unpaired *t*-test.

The species *A. baumannii* presents a distinctive cellular phase-variation. Cells can be subdivided into opaque (VIR-O) or translucent (AV-T) subpopulations which differ with respect to cellular morphology, antibiotic resistance, and virulence in animal models (Tipton et al., 2015; Tipton and Rather, 2017). Fortunately, neither gallein treatment, nor PPK1 knockout influenced the growth kinetics of strains in either phase-variant population (Figures 2E,F and Supplementary Table 2). We therefore sought to determine whether polyP biosynthesis was consistent between individual phase-variants. Both VIR-O and AV-T variants of the parent strain accumulated non-significantly different levels of polyP but were attenuated upon addition of gallein (Figure 3A). When compared with the knockout strains, however, there are notable discrepancies between each phase-variant. The AV-T variant of the ΔPPK1A strain synthesized significantly more polyP than VIR-O (Figure 3A), in contrast to the ΔPPK1B strain, in which the opposite trend was observed (Figure 3A). Despite these relative deviations in polyP accumulation, both phase-variant specific populations adhere to the same relative trend observed with mixed-phase cultures in Figure 2A. In the case of both the VIR-O and AV-T segregated cellular populations, both the parent and ΔPPK1A strains each accumulated significantly more polyP than the ΔPPK1B strain (Figures 3B,C). This phase-variant specific deviation in relative polyP accumulation led us to hypothesize that there were discrepancies in PPK1A and PPK1B transcriptional regulation. We therefore performed qRT-PCR to assess transcript abundance of both genes in the parent and both mutant strains. All transcript abundance trends are qualitatively reported in Table 1. In the case of *ppk1A*, no significant changes in relative expression were observed between phase-variants for the parent and ΔPPK1B strains in logarithmic phase or following nutrient starvation (Figures 3D,E). In the case of *ppk1B*, however, the parent strain AV-T phase-variant significantly upregulated gene expression with respect to the VIR-O during logarithmic phase and following nutrient starvation, though the same trend of increased *ppk1B* transcript abundance was only observed in the ΔPPK1A strain post starvation (Figures 3D,F). We did not observe any changes to *ppk1* transcriptional abundance following treatment with 100 μM gallein during logarithmic phase or post phosphate starvation (Supplementary Figure 6).

**TABLE 1:** Summary of *ppk1* transcriptional trends in the AV-T phase-variant vs. the VIR-O phase-variant of the corresponding condition.

	AB5075	ΔPPK1A	ΔPPK1B
**Gene**			

**0 h—prior to MOPS incubation**			

*ppk1A*	n.s	−	n.s
*ppk1B*	Increased	n.s	−

**2 h—post MOPS incubation**			

*ppk1A*	n.s	−	n.s
*ppk1B*	Increased	Increased	−

*n.s, not significant.*

### Gallein Treatment Reduces Virulence in *A. baumannii*

**FIGURE 4:**
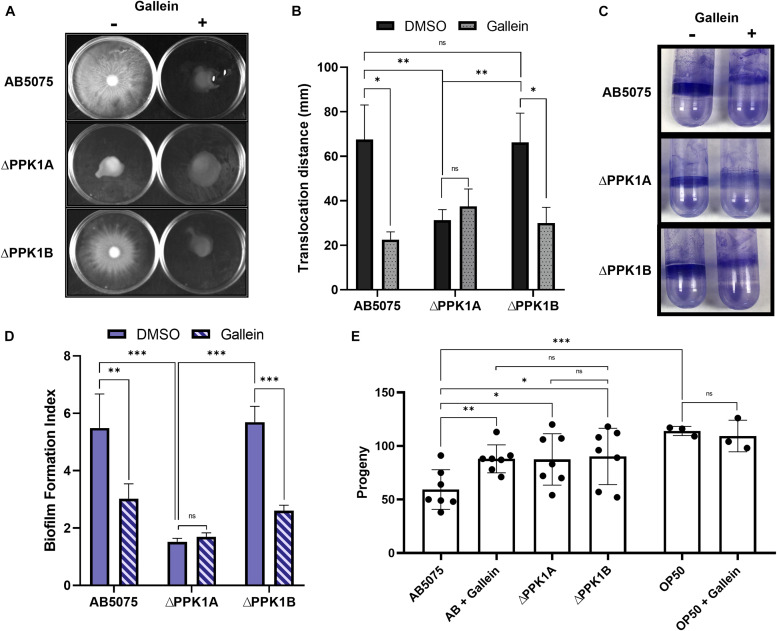
Gallein phenocopies PPK1 knockouts to attenuate *A. baumannii* virulence. **(A)** Representative image of 100 μM gallein treatment and PPK1 knockout on *A. baumannii* surface-associated motility. Image is representative of *n* = 4 assays. **(B)** Quantification of gallein treatment and PPK1 knockout on surface-associated motility. All gallein samples and ΔPPK1A DMSO are not significantly different. **(C)** Representative image of 100 μM gallein treatment and PPK1 knockout on *A. baumannii* biofilm formation. Image is representative of *n* = 3 assays. **(D)** Quantification of gallein treatment and PPK1 knockout on biofilm formation. **(E)** Progeny count of *C. elegans* following feeding with *A. baumannii* strains (*n* = 7) or OP50 (*n* = 3). Symbols are as follows: ns, *p* > 0.05; ^∗^*p* < 0.05; ^∗∗^*p* < 0.01; ^∗∗∗^*p* < 0.001. For **(B,D)** two-way ANOVA, Tukey’s multiple-comparison test; **(E)** two-way Unpaired Welch’s *t*-test. Data points are the average of triplicates or more; error bars are ± SD.

We next attempted to characterize the influence of gallein on *A. baumannii* virulence factors. The VIR-O and AV-T phase-variants are specific for surface associated motility and biofilm formation, respectively, with either phase subpopulation preferentially upregulating the corresponding virulence phenotype (Tipton et al., 2015; Supplementary Figure 7). It is therefore of considerable importance to selectively assay phase-segregated cellular populations when studying *A. baumannii* virulence factors, or risk irreparably confounding findings. For this reason, VIR-O cells were selected for surface associated motility on low-percentage semi-solid media. Under these conditions, both the parent and ΔPPK1B strains translocated significantly farther across the air-media interface than the ΔPPK1A strain (Figures 4A,B). Furthermore, gallein treatment reduced the translocation diameter of the parent and ΔPPK1B strains, while phenocopying the PPK1A knockout mutant with no additive effects (Figures 4A,B). Similar trends were observed for biofilm formation capability using AV-T phase-variant cells. Both the parent and ΔPPK1B strains formed robust biofilms at the air-liquid interface, while the ΔPPK1A strain formed significantly less (Figures 4C,D). In addition, gallein treatment once again reduced biofilm formation of the parent and ΔPPK1B strains while phenocopying the virulestrain, with no additive influence upon total biological material adhered to the abiotic surface (Figures 4C,D). Finally, to assess virulence of *A. baumannii*, a *C. elegans* fertility assay was conducted to quantify the reproductive capability of adult nematodes following bacterial challenge. A recent study (Ahmad et al., 2019) demonstrated that the AB5075 AV-T cellular subpopulation exerts a stronger effect on *C. elegans* fertility, and as such translucent cells were chosen for virulence evaluation. Feeding of both PPK1 knockout strains significantly increased mean nematode progeny with respect to the parent strain (Figure 4E). In turn, gallein treatment significantly increased mean progeny counts to levels seen in PPK1 knockout strains (Figure 4E). Notably, gallein did not influence mean progeny counts on *E. coli* OP50 seeded negative control plates, in keeping with previous observations demonstrating a lack of gallein induced toxicity (Neville et al., 2021).

### Gallein Treatment Does Not Influence a Complex Bacterial Population Resembling Gastrointestinal Microbiota

**FIGURE 5:**
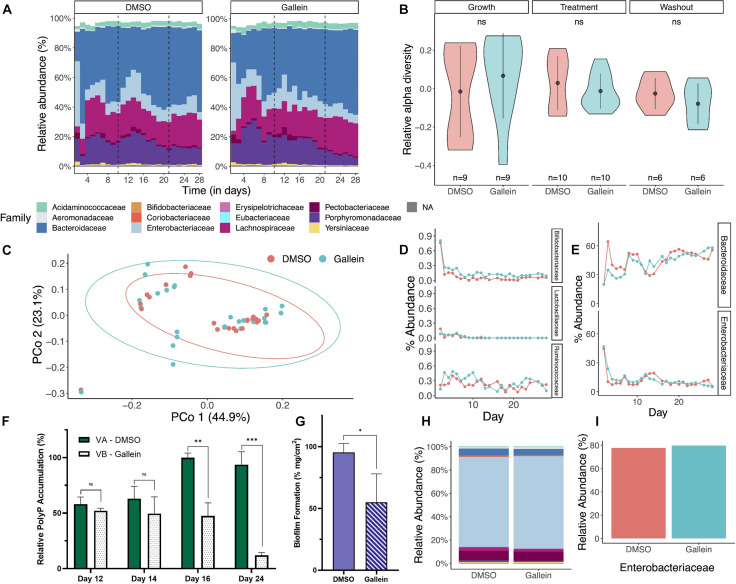
Gallein does not reduce microbial viability in a chemostat model despite metabolically influencing cellular populations. Metagenomic analysis of 16S rRNA sequencing data shows bacterial diversity in chemostats treated with gallein vs. DMSO vehicle over time (growth, treatment, washout phase). **(A)** Relative abundance at the family taxonomic level demonstrates similar composition and distribution following treatment with DMSO or 100 μM gallein. Taxa with a relative abundance less than 5% are combined in white space at the top of each column. **(B)** A violin plot depicting the relative alpha diversity by phase and condition shows no significant difference when comparing condition and phase (Mann–Whitney *U*-test, p_growth_ = 0.2973, p_treatment_ = 0.9705, p_washout_ = 0.5887). Markers and whiskers showing mean ± SD **(C)** Principal coordinate analysis (PCoA) ordination using Bray-Curtis distance showed no distinct clustering by treatment (PERMANOVA: R2 = 0.01823, *p* = 0.44). **(D,E)** Percent relative abundance at the family taxonomic level over the experimental time course for representative Gram positive and Gram negative bacteria, respectively. **(F)** Quantification of total bacterial polyP on select days in DMSO control Vessel A (VA) and gallein treated Vessel B (VB). **(G)** Quantification of adhered dry biological mass per cm^2^ of waste outflow tubing for each chemostat vessel. **(H)** Metagenomic analysis of 16S rRNA sequencing demonstrates a similar relative abundance of bacteria at the family taxonomic level in the biological material adhered to the waste outflow tubing of each chemostat vessel. **(I)** Percent relative abundance of bacteria belonging to the *Enterobacteriaceae* family within the biological material adhered to the chemostat waste outflow tubing. Symbols are as follows: ns, *p* > 0.05; ^∗^*p* < 0.05; ^∗∗^*p* < 0.01; ^∗∗∗^*p* < 0.001 (two-way Unpaired *t*-test). For **(F)** data points are the average of triplicates; for **(G)** data points are generated from the average of three sections of tubing; error bars are ± SD.

Next, we sought to assess the non-disruptive properties of gallein which remain a hallmark of anti-virulence therapeutics. Chemostat bioreactors are used to culture complex microbial communities that resemble those within the gastrointestinal microbiota (GIM). These systems employ a continuous culture model and provide valuable insight into compositional changes in microbial communities over time and in response to perturbations (Guzman-Rodriguez et al., 2018). To assess the effects of gallein on the GIM, a twin-vessel chemostat system was inoculated with 33 different bacterial strains, representative of a “healthy” GIM community. The chemostats showed similar changes in microbial distributions and compositions at the family taxonomic level across time (Figure 5A). We found no significant difference in relative alpha diversity when comparing treatment conditions within a given phase or when comparing phases within a given treatment (Figure 5B). There is no significant difference in beta diversity, as can be seen in PCoA clustering when comparing treatment groups (Figure 5C) and validated using Jaccard’s distance. We observed that time had a significant effect on alpha and beta diversity (Supplementary Figure 8), but these changes were consistent in both treatment and control. We did not observe differences in the percent abundance of selected families of Gram positive (*Bifidobacteriaceae, Lactobacilliaceae*, and *Ruminococcaceae*) organisms and Gram negative (*Bacteroidaceae* and *Enterobacteriaceae*) organisms (Figures 5D,E). The addition of gallein was found to significantly reduce total community polyP abundance during the treatment time course, as well as following compound washout (Figure 5F). Interestingly, we also observed a significant reduction in adhered exopolymer within the waste outflow tubing of the chemostat vessel which had received gallein treatment, compared to DMSO (Figure 5G). When evaluating the community composition of the adhered biological matrices, we did not notice any significant differences between the vessel that was exposed to gallein compared to the vessel exposed to DMSO (Figure 5H). Furthermore, when assessing percent abundance of bacteria within the family *Enterobacteriaceae* specifically, we did not notice a difference between the two vessels (77.7 and 79.7%) (Figure 5I).

## Discussion

PPK knockout strains of pathogenic bacteria have long been known to harbor defects in stress response mechanisms and virulence factor pathways (Fraley et al., 2007; Gangaiah et al., 2010; Chuang et al., 2013; Batten et al., 2016; Dahl et al., 2017). This decades old proof of concept has unsurprisingly enticed researchers to develop inhibitors targeting bacterial PPKs (Singh et al., 2016; Dahl et al., 2017; Bashatwah et al., 2018; Burda-Grabowska et al., 2019). Although several inhibitors have been identified, these molecules suffer from their inability to selectively inhibit multiple PPK isoforms. Moreover, seldom are these molecules assessed for broad-spectrum activity in a variety of bacteria (Bowlin and Gray, 2021). We recently demonstrated that the small molecule gallein had dual-specificity inhibition of all four *P. aeruginosa* PPK enzymes while avoiding off-target toxicity (Neville et al., 2021).

These observations formed the premise for the current work, to determine whether gallein was capable of broad-spectrum PPK inhibition across multiple priority pathogens. The species *A. baumannii* and *K. pneumoniae* were chosen as they currently are both considered critical priority pathogens by the WHO (World Health Organization, 2017). Furthermore, the body of literature pertaining to polyP or PPK mediated virulence of these species is lacking. Recently, inhibitors of *A. baumannii* PPK1B were identified from repurposed drugs (Gautam et al., 2021), marking the first advancement in *A. baumannii* PPK research in several years. However, there was a lack of control for the characteristic phase-variation of this species when assessing virulence factors. Given the stark phenotypic disparity of each phase-variant subpopulation (Tipton et al., 2015; Supplementary Figure 7), this leaves any assessment of drug induced attenuation intrinsically confounded, and most conclusions thereof subject to scrutiny.

Consistent with *P. aeruginosa*, gallein treatment successfully inhibited PPK1s *in vitro* from multiple species of *Enterobacteriaceae* (Figures 1B,C) as well as PPK2 from *K. pneumoniae* (Supplementary Figure 2). *K. pneumoniae* intracellular polyP accumulation was also significantly reduced (Figure 1D), which in turn resulted in the attenuation of biofilm formation (Figures 1E,F). Adhered exopolymer was also significantly reduced in the chemostat vessel waste outflow junction which had received gallein (Figure 5G). Sequencing revealed that the biological matrix was overwhelmingly composed of species belonging to the *Enterobacteriaceae* family, at an equivalent abundance (Figures 5H,I). This reduction in *Enterobacteriaceae* adhered exopolymer is thus consistent with *in vitro* biofilm attenuation following gallein treatment of *K. pneumoniae* monocultures (Figure 5F), with no changes to cellular growth kinetics (Figure 1G). To our knowledge, these findings represent the first evidence of selective drug targeting and anti-virulence inhibition of *K. pneumoniae* PPKs.

While previous studies have characterized PPK1 enzymes from *Acinetobacter* sp. (Gavigan et al., 1999; Trelstad et al., 1999), these focused on the PPK1B isoform. *A. baumannii* presents with a rare instance of encoding a second putative PPK1—namely PPK1A. Both PPK1A, PPK1B, as well as PPK2 from *A. baumannii* were inhibited by gallein treatment (Figures 2C,D and Supplementary Figure 2, respectively), though the kinetic parameters between both PPK1 enzymes were not consistent. PPK1A maximal activity *in vitro* (Figure 2C) was several-fold lower than that of PPK1B (Figure 2D). These experimental findings indicate discrepancies at the biochemical level between PPK1A and PPK1B, which is a somewhat surprising implication given the enzymes share ∼67% sequence identity (Supplementary Figure 1). While more thorough characterization is still required, our molecular dynamics simulations suggest some difference in secondary structure conformation and residue interactions along the homodimerization interface (Supplementary Figures 9, 10). It therefore remains a possibility that *A. baumannii* PPK1A and PPK1B functional variations are a result of deviations in oligomeric contacts, which have been shown to affect the activity of *E. coli* PPK1 (Tzeng and Kornberg, 2000; Rudat et al., 2018). It is tempting to speculate that an additional level of enzymatic regulation carries with it broader niche-specific physiological significance, as is often the case with species encoding multiple PPK2 isoforms (Zaborin et al., 2009; Tumlirsch et al., 2015; Racki et al., 2017; Neville et al., 2021).

PolyP accumulation in individual knockout strains of either enzyme indicates that intracellular biosynthesis is mediated primarily through PPK1B activity (Figure 2A). PolyP accumulation in the ΔPPK1A strain was unaffected, yet the ΔPPK1B strain had significantly reduced levels of intracellular polyP (Figures 2A,B), consistent with weaker PPK1A activity *in vitro* (Figures 2C,D). All three strains were significantly attenuated in polyP accumulation following gallein treatment (Figure 2A), in accordance with the enzymatic inhibition observed *in vitro*. While it cannot be fully ruled out that *A. baumannii* PPK2 is not responsible for some degree of intracellular polyP accumulation, polyP biosynthesis mediated by class 2 PPK2 enzymes from other species is negligible and insufficient for polyP granule biogenesis (Racki et al., 2017).

Further functional variations between PPK1A and PPK1B are observed for intracellular polyP accumulation following phase-variant segregation, as single knockout strains favored polyP biosynthesis in particular subpopulations (Figure 3A). Transcriptional variation was partially responsible for this phenomenon, and a summary of relative trends is shown in Table 1. Both the parent and ΔPPK1A strain displayed an increased expression of *ppk1B* in the AV-T phase-variant when compared to the VIR-O following phosphate starvation (Figures 3D,F). It therefore follows that the greater accumulation of polyP observed in the ΔPPK1A strain AV-T cells (Figure 3A) is likely due to elevated phase-specific *ppk1B* transcription. In the case of *ppk1A*, however, this is inconsistent. While it has been shown that PPK transcript abundance increases in *Acinetobacter* sp. following phosphate deprivation and induction of the *pho* regulon (Gavigan et al., 1999; Santos-Beneit, 2015), we did not detect any significant phase-variant specific *ppk1A* transcriptional upregulation under the assayed conditions in the parent or ΔPPK1B strains (Figures 3D,E).

The reduction in polyP accumulation observed in the ΔPPK1B AV-T cellular phase (Figure 3A) was therefore not a result of selective *ppk1A* transcriptional upregulation in VIR-O cells as originally anticipated. There could be many possible explanations; for example, biased transcription or activation of polyP biosynthesis antagonists such as PPK2 or exopolyphosphatase (PPX) (Akiyama et al., 1993) in AV-T cells. Alternatively, possible protein binding partners (Rudat et al., 2018), or alternative transcriptional regulatory elements (Gray, 2019) could instead serve to modulate PPK1A activity more stringently.

Surprisingly, the *A. baumannii* ΔPPK1A strain was significantly attenuated in surface-associated motility (Figures 4A,B). This was consistent with reports of motility deprivation in other *ppk* knockout species (Rashid et al., 2000; Fraley et al., 2007), and despite *A. baumannii* being non-flagellated, it does encode type IV pili required for twitching motility (Harding et al., 2013). This phenotype is PPK1 dependent in *P. aeruginosa* (Rashid and Kornberg, 2000), and these molecular structures appear to be at least partially responsible for *A. baumannii* surface-associated motility (Clemmer et al., 2011). There are, however, conflicting reports on the full involvement of type IV pili during surface-associated motility (Clemmer et al., 2011; Harding et al., 2013). While the ΔPPK1A strain was significantly attenuated in cellular translocation, the ΔPPK1B strain was not (Figures 4A,B), despite the VIR-O phase-variant accumulating significantly less polyP (Figure 3B). Similar trends were observed for *A. baumannii* biofilm formation. Once again, the ΔPPK1A AV-T phase-variant accumulated comparable levels of polyP to that of the parent (Figure 3C) yet demonstrated a significant reduction in biofilm formation (Figures 4C,D). Interestingly, biofilm formation was also found to be influenced by fewer cellular passages (Supplementary Figure 11). This could hint at more complex PPK homeostatic regulation over the course of the bacterial lifecycle. Nevertheless, gallein treatment phenocopied the PPK1A knockout in both the parent and ΔPPK1B strains after passaging (Figures 4B,D), with no additive effects, suggesting that attenuation of both phenotypes under the assayed conditions is indeed PPK1A dependent. These are important findings, as discussed both ΔPPK1A phase-variants are not attenuated in polyP biosynthesis compared to the parent (Figures 3B,C). This would suggest that virulence phenotypes are less reliant upon increased intracellular polyP stores, as is generally the case in other species (Shiba et al., 1997; Dahl et al., 2017; Neville et al., 2021). In *A. baumannii*, intracellular polyP reserves alone are insufficient for phenotype presentation, but rather PPK1A function is necessary. Taken together, this could suggest that PPK1A and PPK1B possess unique intracellular functional or regulatory roles beyond polyP biosynthesis. In addition, the PPK1A isoform would appear to be more closely associated than PPK1B with the mechanisms of virulence-factor presentation.

Virulence of both knockout strains as well as the parent strain of *A. baumannii* treated with gallein was significantly attenuated in a *C. elegans* fertility model. Lower progeny counts correlate with increased colonization susceptibility of the nematodes (Vallejo et al., 2015), and in each case, mean progeny counts were significantly higher than that of the parent (Figure 4E). Notably, the ΔPPK1B strain was deficient in virulence as well, suggesting that despite showing no defects in motility and biofilm formation, it nevertheless is attenuated in some element of colonization. Given the ΔPPK1B strain accumulates significantly less polyP, one possibility, is perhaps the observed suppression of virulence is a result of an inability to adequately respond to physical stressors (Rao and Kornberg, 1996). *C. elegans* comprises “ancestral” immunity (Pukkila-Worley and Ausubel, 2012; Ermolaeva and Schumacher, 2014), and while certainly not as robust as the mammalian immune system, still employs the generation of cytotoxic reactive oxygen species (Chávez et al., 2007), synthesis of antimicrobial peptides by intestinal epithelial cells, and the ability to mount pathogen-specific immune responses (Pukkila-Worley and Ausubel, 2012). Long chain polyP has been shown in *E. coli* and *P. aeruginosa* to play an essential role as a phosphate nutrient reservoir, as well as a determinant in tolerance to oxidative stress, osmotic shock, and survival during stationary phase (Rao and Kornberg, 1996; Shiba et al., 1997; Gray et al., 2014; Dahl et al., 2017). It therefore stands to reason that failure to synthesize sufficient polyP could result in reduced bacterial physical stress tolerance during acute infection.

We did not observe notable differences between the chemostat vessel that was exposed to gallein compared to the one exposed to DMSO vehicle. There were no differences in species richness (alpha diversity), species diversity (beta diversity) or taxonomic distributions when comparing chemostat vessels (Figures 5A–E). These findings are important because they suggest that not only does gallein attenuate virulence factors of *K. pneumoniae* and *A. baumannii* but does so without damaging the GI bacterial flora, a side effect of current antibiotic therapies (Francino, 2016). Notably, gallein treatment reduced total community polyP accumulation, without hindering community fitness (Figure 5F).

It must be considered, however, that the chemostat model does not fully encapsulate the complexity of the human gut microbiome. Interactions of the GIM with the host are important considerations both in the context of microbial diversity and gastrointestinal health (Jia et al., 2008). It is also worth noting, that gallein has been independently identified as a G-protein βγ subunit antagonist (Smrcka, 2013), further implicating the propensity for off-target effects in humans. In our previous work, however, we demonstrated that gallein exhibited no detectable toxicity toward cultured mammalian cells (Neville et al., 2021). These findings were consistent with studies in which elevated doses of gallein were administered to mice or rats for up to 8 weeks with no adverse effects (Kamal et al., 2014; Sanz et al., 2017; Karuppagounder et al., 2018). Likewise, bacterial long chain polyP has also recently been shown to suppress pro-inflammatory signaling cascades of the innate immune system (Roewe et al., 2020). It is therefore an intriguing notion that perhaps a dual-specificity inhibitor of polyP biosynthesis could serve to enhance host innate immunity and thus indirectly aid in clearing pathogens during infection *in vivo*.

Gallein treatment has now been shown in this work to act as a broad-spectrum inhibitor attenuating virulence of *K. pneumoniae*, and *A. baumannii* in addition to *P. aeruginosa* (Neville et al., 2021), while remaining innocuous to beneficial human microflora. Moreover, gallein treated controls aided in the identification of novel *A. baumannii* PPK1 physiological elements, highlighting the unique prospect of employing this inhibitor as a tool to study bacterial polyP homeostasis. Together, these works serve to lay the foundations for developing a novel class of anti-virulence therapeutic, whilst paving the way for new discoveries in the field of polyP research.

## Data Availability Statement

The datasets presented in this study can be found in online repositories. The names of the repository/repositories and accession number(s) can be found below: www.ncbi.nlm.nih.gov/, PRJNA758409.

## Author Contributions

NR, NN, and ZJ performed all *in vitro* and bacterial virulence experiments. KD, CN, and PS assisted in the planning of and execution of the chemostat experiment. NB and CS analyzed chemostat sequence data and generated the corresponding figures. KD and CS wrote the manuscript sections pertaining to the chemostat methodology and experimental analysis, NR wrote the remaining manuscript. All authors analyzed the data, designed the experiments, provided edits, read, and approved the final version of the manuscript.

## Conflict of Interest

The authors declare that the research was conducted in the absence of any commercial or financial relationships that could be construed as a potential conflict of interest.

## Publisher’s Note

All claims expressed in this article are solely those of the authors and do not necessarily represent those of their affiliated organizations, or those of the publisher, the editors and the reviewers. Any product that may be evaluated in this article, or claim that may be made by its manufacturer, is not guaranteed or endorsed by the publisher.

## Footnotes

1
https://www.ncbi.nlm.nih.gov/nuccore/NZ_CP008827.1

2
https://www.ncbi.nlm.nih.gov/nuccore/NZ_CP008706.1

3
www.ncbi.nlm.nih.gov/tools/primer-blast/

4
https://github.com/benjjneb/dada2

5
https://www.gs.washington.edu/labs/manoil/
