# Supplementary material for: Broad-Spectrum Inhibitor of Bacterial Polyphosphate Homeostasis Attenuates Virulence Factors and Helps Reveal Novel Physiology of Klebsiella pneumoniae and Acinetobacter baumannii
Source: Front Microbiol. 2021 Oct 26;12:764733. doi: 10.3389/fmicb.2021.764733 (PMC8576328; doi:10.3389/fmicb.2021.764733)
Supplement: Supplementary file 1 [file Data_Sheet_1.PDF]

Supplemental Information

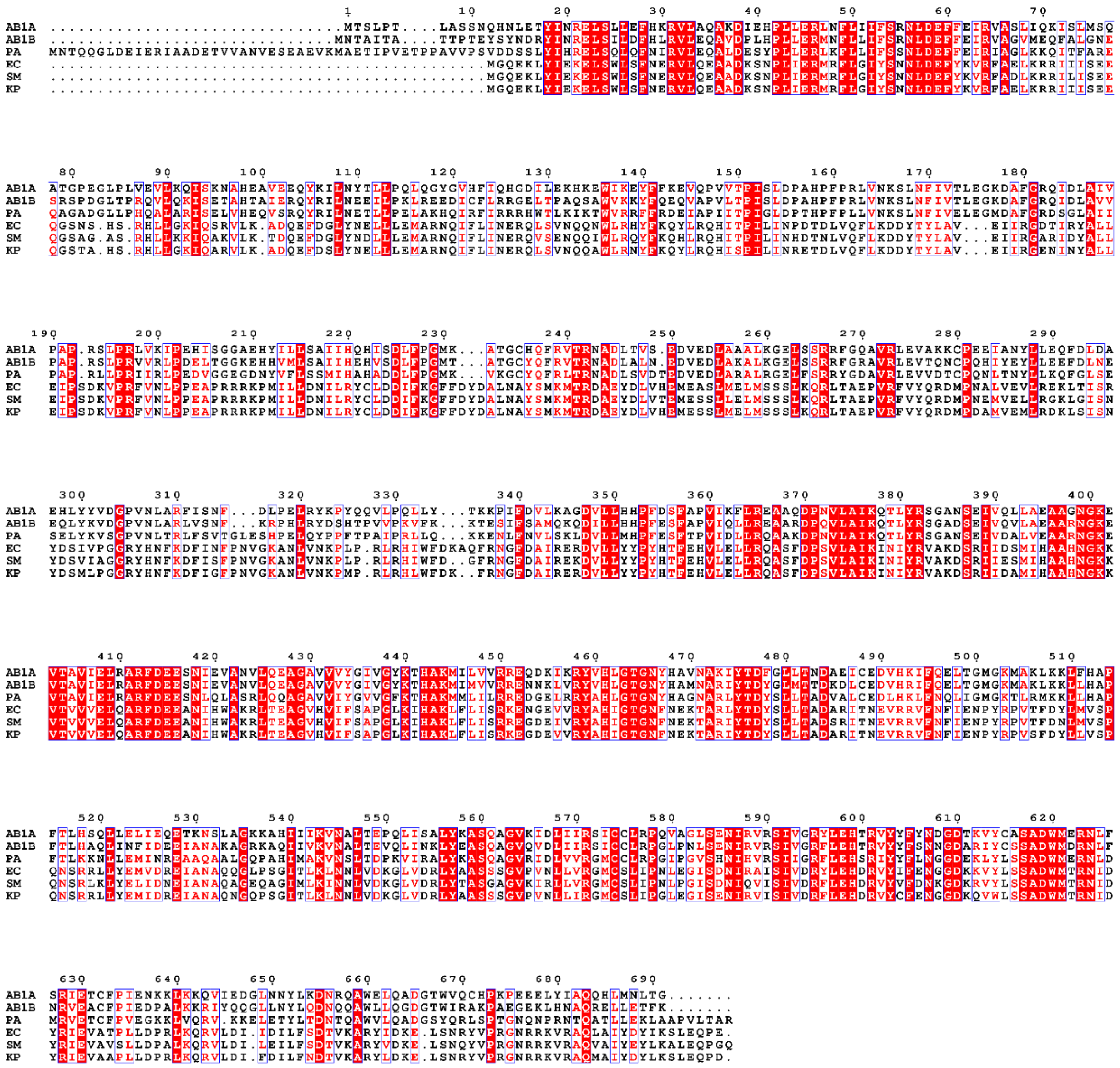

Fig. S1: Sequence alignment of Gram negative bacteria PPK1 enzymes. Sequence alignment of all PPK1s assessed in this study; *Acinetobacter baumannii* PPK1A (AB1A), *Acinetobacter baumannii* PPK1B (AB1B), and *Pseudomonas aeruginosa* PPK1 (PA), *Escherichia coli* (EC), *Serratia marcescens* (SM), and *Klebsiella pneumoniae* (KP). *A. baumannii* PPK1A and PPK1B sequence identity: 67.44%. Red shading indicates identical residues, and blue borders indicate partially conserved residues. Sequence alignment generated using ESPript 3.0 (Robert and Gouet 2014).

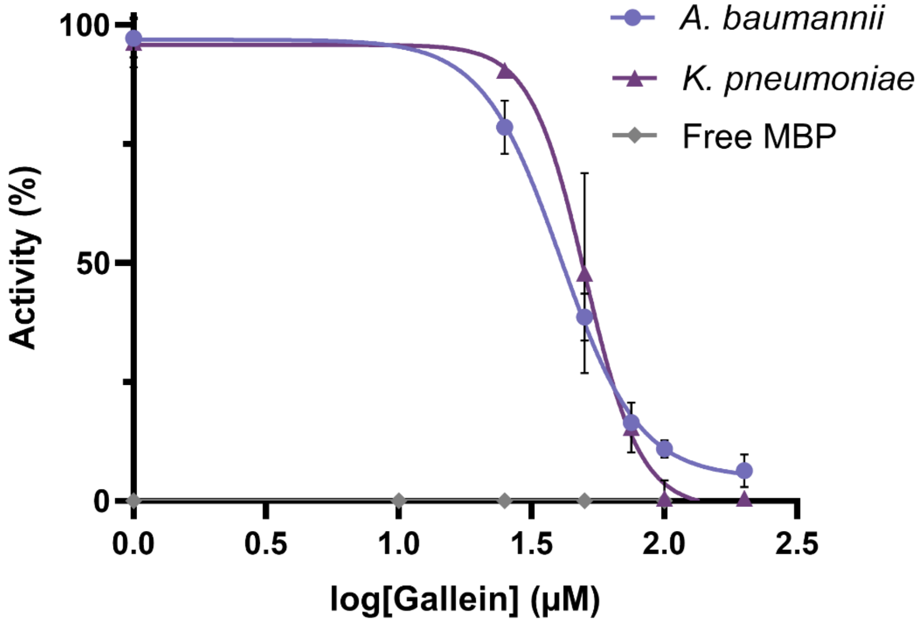

Fig. S2: Dose-dependent inhibition of purified PPK2 catalyzed ADP (*A. baumannii*) and ATP (*K. pneumoniae*-MBP fusion) synthesis from polyP degradation. Data points are presented as the mean, error bars are ±SD

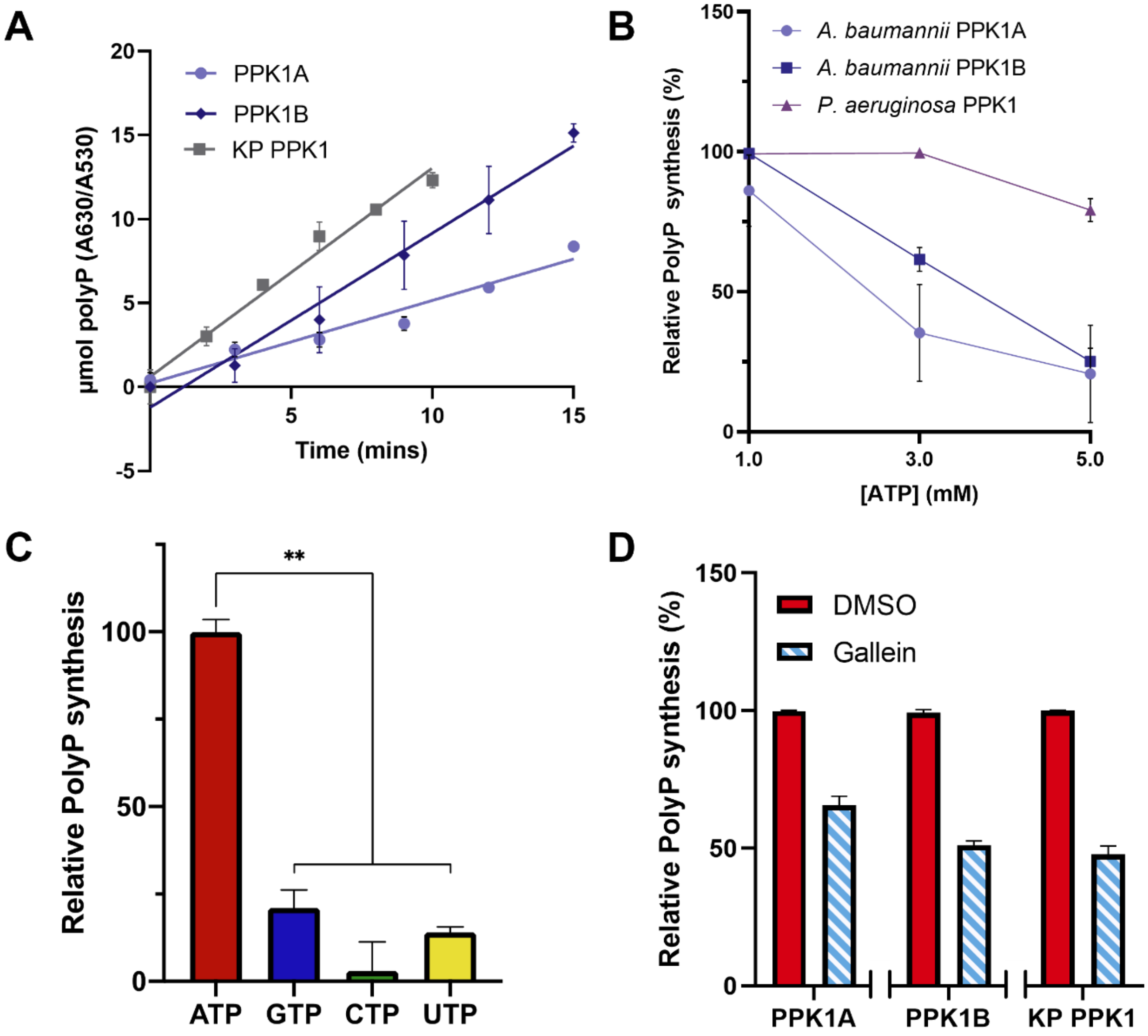

Fig. S3: Enzymatic properties of recombinantly expressed and purified PPK1 enzymes. (A) Representative linearization of *in vitro* polyP synthesis by purified PPK1 at 1 mM ATP. **(B)** Substrate inhibition at [ATP] above 1 mM, with respect to *P. aeruginosa* PPK1 (Neville et al. 2021). **(C)** Nucleotide specificity of *A. baumannii* PPK1A without creatine kinase catalyzed nucleotide regeneration: **, $p < 0.01$ (two-way Unpaired t test, $n = 2$). **(D)** Relative polyP synthesis in the presence and absence of 100 µM gallein or equivalent volume of DMSO and without creatine kinase catalyzed ATP regeneration. Data points are presented as the mean, error bars are ±SD

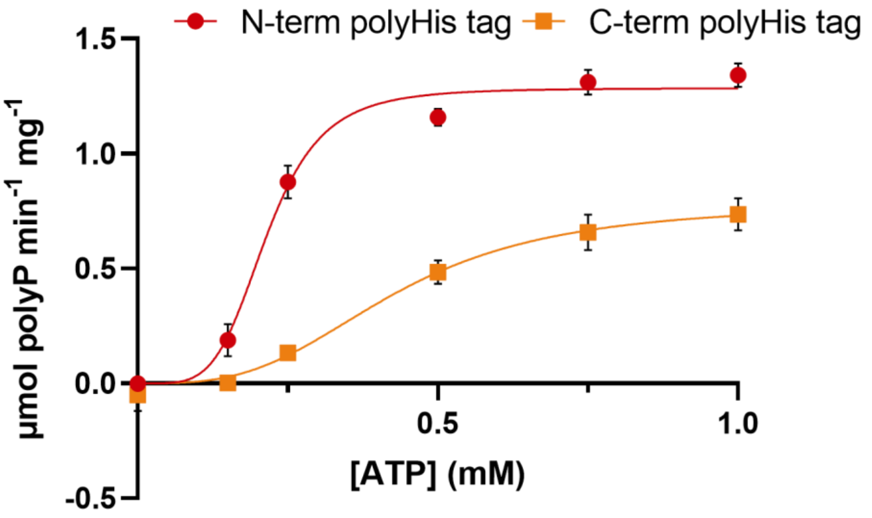

Fig. S4: Terminally His$_6$-tagged PPK1B activity comparison. Specific activity of N or C-terminally His$_6$-tagged protein. Data points are presented as the mean, error bars are ±SD.

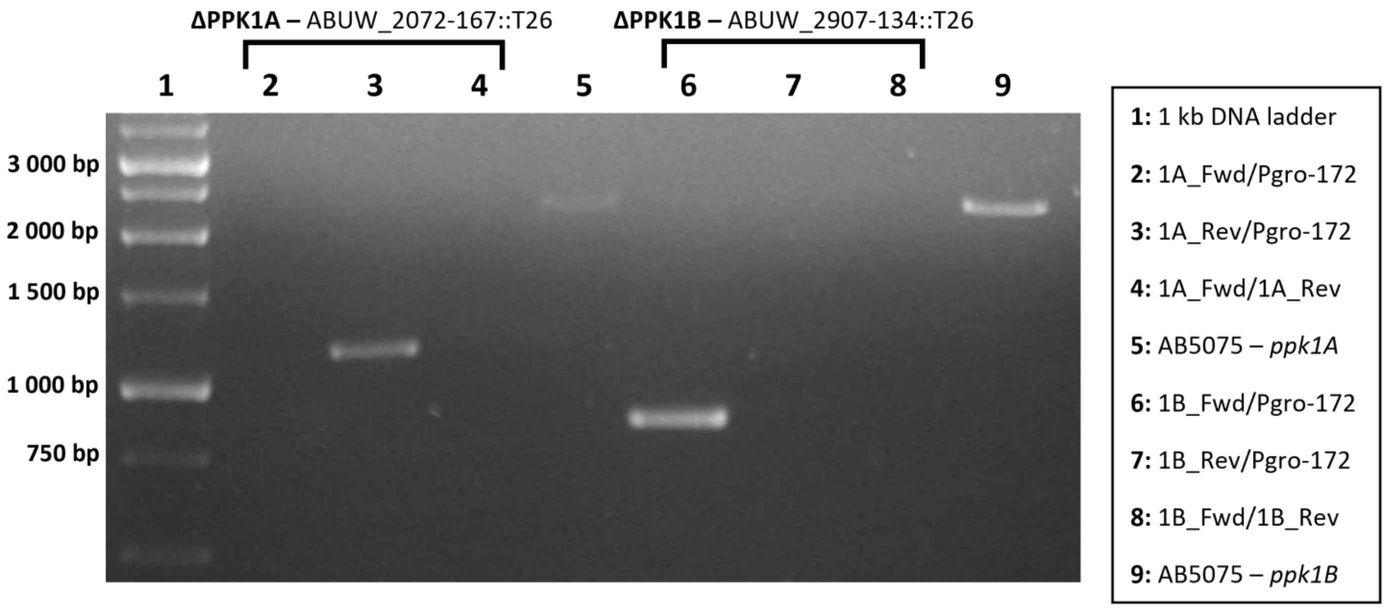

Fig. S5: *A. baumannii* transposon mutant confirmation. Presence of single bands in lanes 3 and 6 indicate successful directional T26 transposon insertion into *ppk1A* (1A) and *ppk1B* (1B) respectively.

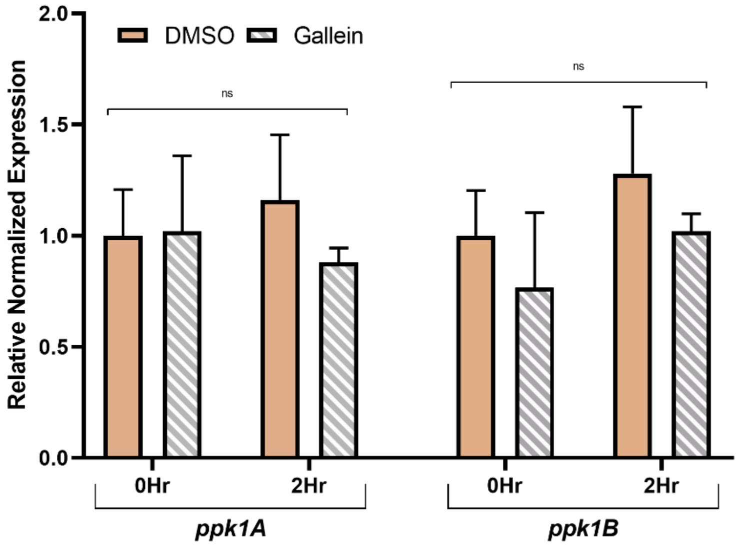

Fig. S6: Gallein does not influence *A. baumannii ppk1* transcription. Relative normalized expression of *ppk1A* and *ppk1B* in *A. baumannii* parent strain AV-T phase-variant following 2 hours phosphate-starvation. Cultures were treated with 100 µM gallein during logarithmic phase and again during phosphate nutrient starvation. Data points are the averages from 3 independent RNA isolations, error bars are ±SEM. All *ppk1* transcription is not significantly different.

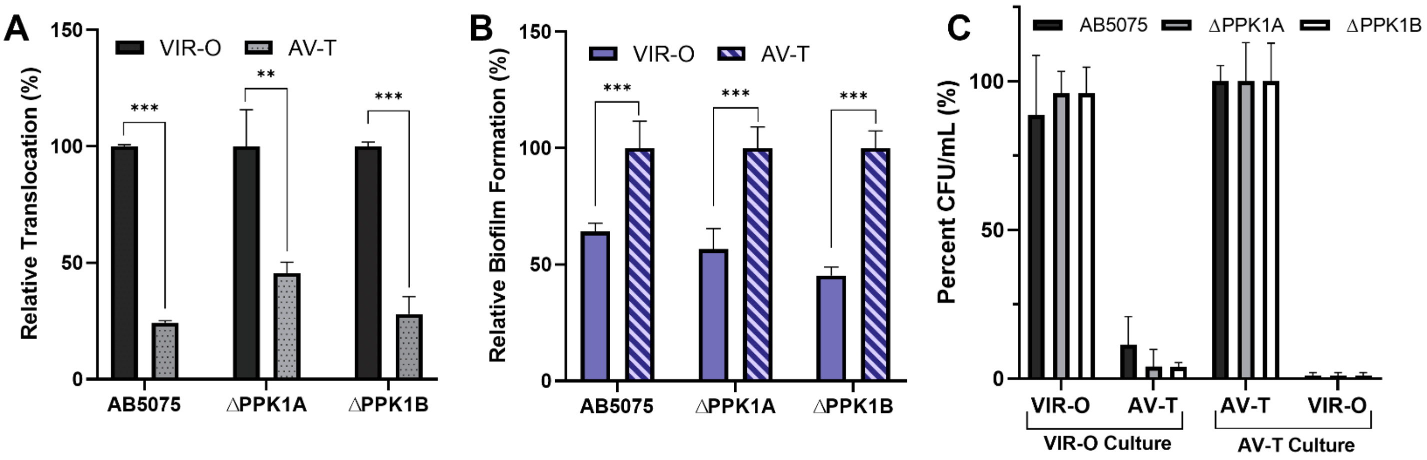

Fig. S7: Relative normalized *A. baumannii* phase-variant subpopulation phenotypes. (A) Relative surface translocation distance of phase-variant subpopulations. Each strain is normalized with respect to individual VIR-O phase. (**B)** Relative biofilm formation of phase-variant subpopulations. Each strain is normalized with respect to individual AV-T phase. **(C)** Representative normalized CFU/mL analysis of phase-variant subpopulations grown overnight in LB media with no NaCl, n =3. For panels A and B, symbols are as follows: **, $p < 0.01$; ***, $p < 0.001$ (two-way ANOVA, Tukey's multiple-comparison test, **(A)** n = 2, and **(B)** n = 8). Data points are presented as the mean, error bars are ±SD

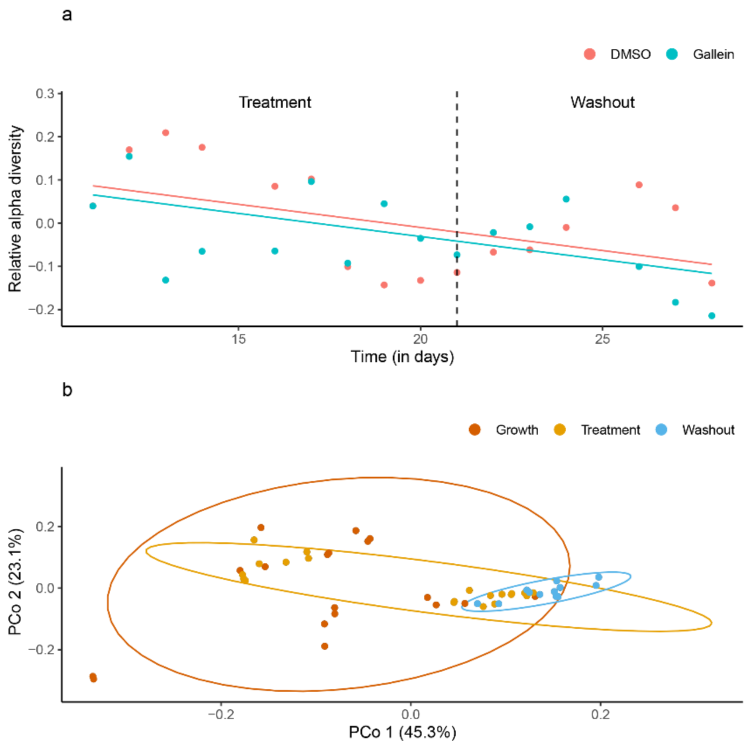

Fig. S8: Changes in diversity across time and phases. (A) Scatter plot and linear mixed model depicting the decrease in relative alpha diversity over time. Reactors were held as a random effect while time had a significant fixed effect ($p = 0.00273$, 95% CI [-0.017, -0.004]). **(B)** PCoA ordination using Bray-Curtis distance showed a distinct tightening of clusters by phase (PERMANOVA, $R2 = 0.21929$, $p < 0.01$).

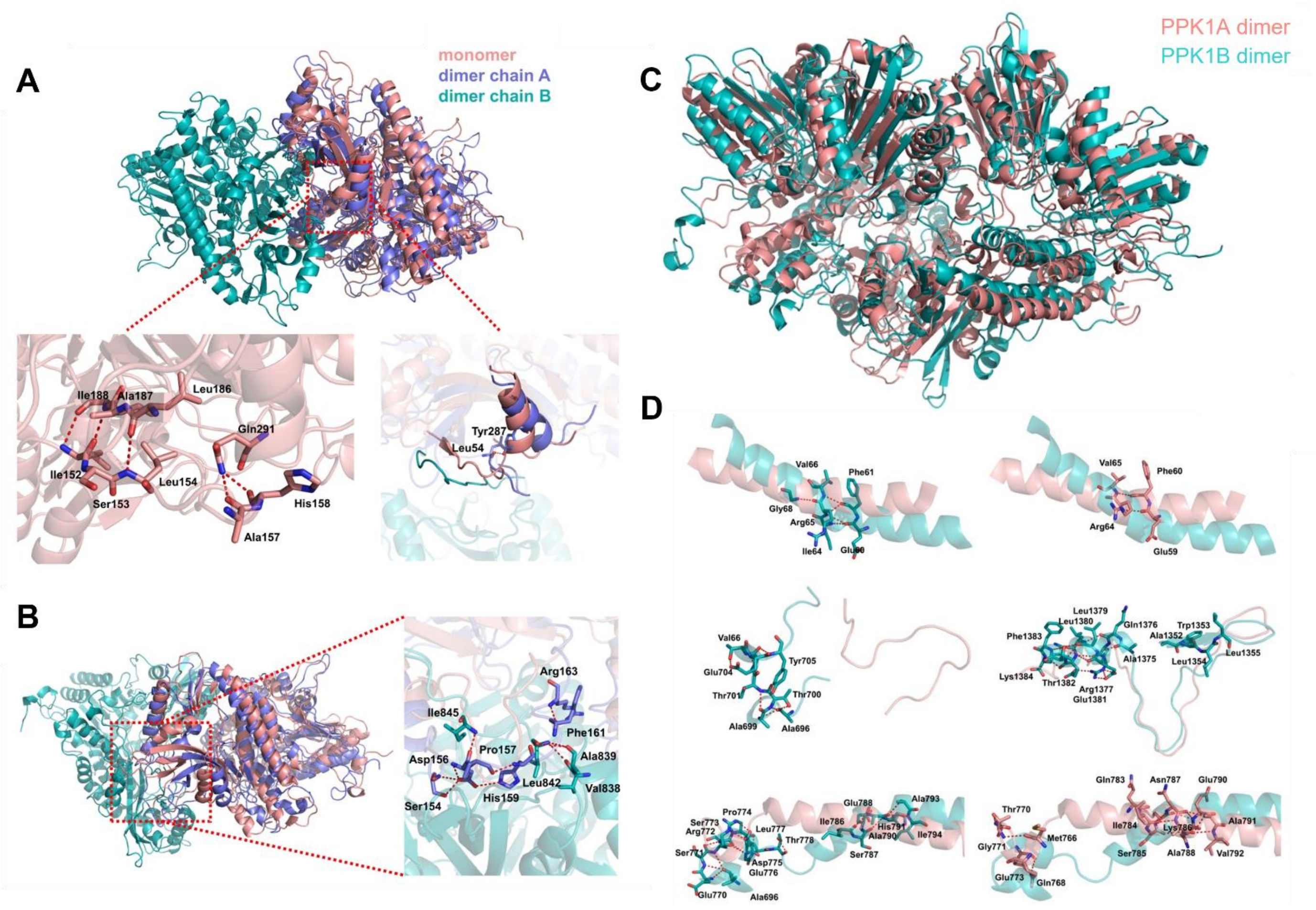

Fig. S9: Molecular dynamics models of PPK1A and PPK1B dimers. (A and B) The structural superimposition in monimeric and dimeric forms of PPK1A and PPK1B respectively. **(C)** The structural superimposition of PPK1A and PPK1B dimers **(D)** The structural superimposition of some residues with conformational differences in PPK1A and PPK1B dimers

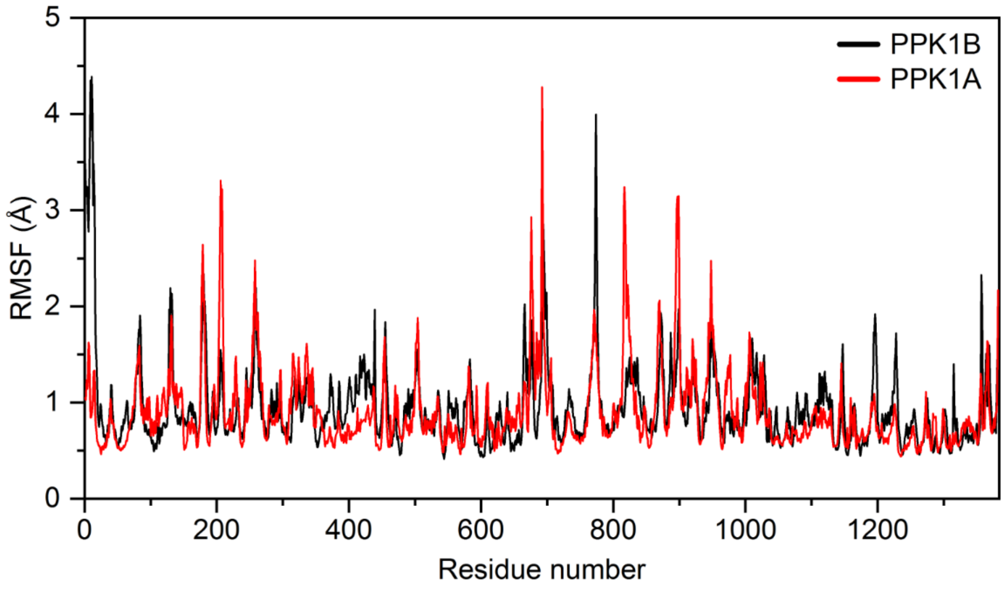

Fig. S10: The fluctuations of residues in the PPK1A dimer (in red) and PPK1B dimer (in black).

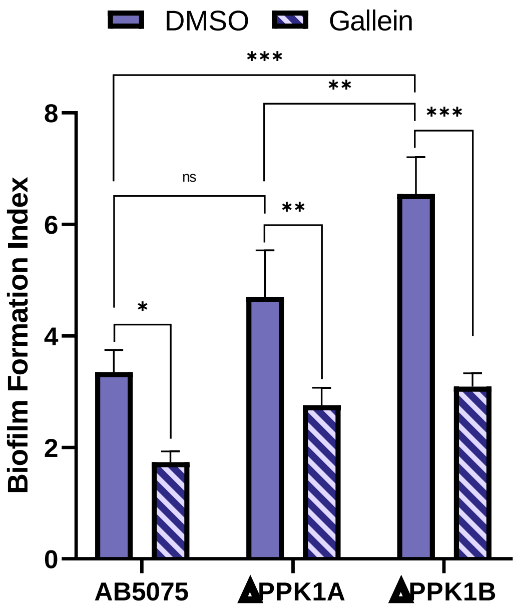

Fig. S11: *A. baumannii* biofilm formation quantification using cells streaked directly from frozen (no cell passages). Symbols are as follows: ns, $p > 0.05$; *, $p < 0.05$; **, $p < 0.01$; ***, $p < 0.001$ (two-way ANOVA, Tukey's multiple-comparison test.) Data points are the average of triplicates; error bars are ±SD.

Supplemental Methods

Molecular dynamics modelling

From previous work with *E. coli* PPK1, the minimum functional oligomerization of this enzyme for the synthesis of polyP is known to be a dimer (Tzeng and Kornberg 2000). Thus, the structures of PPK1A and PPK1B are modelled as dimers, with a dimeric template (PDB CODE 2O8R). The monomer and dimer proteins were then submitted for further molecular dynamics (MD) simulations. The protonation state of all amino acid residues was assigned by using Ambertools. These protein models were then soaked into TIP3P water box with a minimum distance of 10 Å to the protein boundary. Counter ions Na^+ were added in the box to neutralize the system. Each of the processed systems was then submitted to an energy minimization. After that, the systems were then heated to 300 K and equilibrated for 300 ps. Finally, MD simulations were performed in the NPT ensemble for 10 ns to obtain the product phase. Energy minimization, heat and MD simulations were carried out using Amber14 program with the Amber ff14SB force field.

Supplemental Results

Chemostat changes with time

We found a moderately negative correlation between time (following the growth phase) and relative alpha diversity (Spearman's, n= 32, ρ = -0.4669609, p = 0.007052). To determine the effects of gallein during the treatment and washout phases, model selection was conducted with a series of nested linear mixed models. The model that best fit the data was

lmer(relative_alpha ~ Time + (1|chemostat), data=alpha [input$Time>10,]) where time was the sole significant factor (p = 0.00273, 95% CI [-0.017, -0.004]) (Fig S8A) and treatment with gallein or the interaction between time and treatment were not statistically significant.

Temporally, there is a significant difference in dispersion across phases (PERMANOVA, $R2 = 0.21929$, $p < 0.01$) (Fig S8B). To determine the impact of species heterogeneity, we used linear, zero-intercept linear, logarithmic and zero-intercept logarithmic models along with an anova and the minimum AICc; and the logarithmic model best described the observed changes in beta diversity over time (**DMSO**: $R2 = 0.7667$, $F(1,13) = 42.72$, $\beta = 0.4305$, $p = 1.894e-05$, **gallein**: $R2 = 0.8943$, $F(1,13) = 109.9$, $\beta = 0.5484$, $p = 1.035e-07$).

Molecular dynamics simulations of PPK1A and PPK1B reveal differences in oligomeric interactions

To further probe for biochemical discrepancies at the enzymatic level, molecular dynamics (MD) simulations derived from homology models of PPK1A and PPK1B were performed. From previous work with *E. coli* PPK1, the minimum functional oligomerization of this enzyme for the synthesis of polyP is known to be a dimer, though higher order oligomeric states are possible (Tzeng and Kornberg 2000). As such, a dimer was chosen as the functional unit for analysis. From the MD simulations of the dimeric models, the two PPK1 monomers intertwine, with the residues 150-190, and 380-415 comprising the bulk of the protein-protein interactions. With respect to the monomer, several new hydrogen bonds and hydrophobic interactions form at the dimeric interface for both models (Fig S9), which resulted in the distortion of the surface. Though the overall structure of the PPK1A dimer is similar to that of the PPK1B dimer (Fig S9C), there remain subtle discrepancies in secondary structure. To clarify

the conformational changes for the dimer formations, the root-mean-square fluctuations (RMSF) values of each residue were analyzed (Fig S10). The large difference of RMSF values between the two dimers were found mainly in the residues 1-16, 439, 699, 773, 774, 775, 1357, 1358, 1383 and 1384. The residues 1-16 are situated in the loop zone showing flexibility, while the increase of RMSF values in the 439, 699, 773, 774, 775, 1357, 1358, 1383 and 1384 positions is accompanied by different hydrogen bonds and hydrophobic interactions (Fig S9D).

Table S1: Strains, plasmids, and oligos used in this study

Strains

Strain name	Genotype	Source
***E. coli* strains**		
BL21 (DE3)	fhuA2 [lon] ompT gal (λ DE3) [dcm] ΔhsdS λ DE3 = λ sBamHIo ΔEcoRI-B int::(lacI::PlacUV5::T7 gene1) i21 Δnin5	New England Biolabs
OP50	–	Available from CGC, University of Minnesota
***K. pneumoniae* and *A. baumannii* strains**		
KPNIH1	*K. pneumoniae* UW parent strain	Manoil lab library, University of Washington
AB5075	*A. baumannii* UW parent strain	
ΔPPK1A	ABUW_2072-167::T26	
ΔPPK1B	ABUW_2907-134::T26	

Plasmids

Plasmid name	Purpose	Antibiotic	Source
pET28a	PPK1 inducible expression system	Kan^R	This study
HT7	Expression vector based on pET16 with N-His6-TEV-MCS-C	Amp^R	This study
HT29	Expression vector based on pET16 with N-His6-MBP-TEV-MCS-C	Amp^R	(Neville et al. 2021)

Oligos – Cloning

Oligo name	Sequence	Restriction enzyme	Target Gene
EC PPK_fwd	atatCCATGGCAATGGGTCAGGAAAAGCTATAC	NcoI	*E. coli* K12 *ppk1*
EC PPK_rev	atatCTCGAGTTCAGGTTGTTCGAGTGATT	XhoI	
SM PPK_fwd	atatCCATGGCAATGGGTCAGGAAAAGCTCTAC	NcoI	*S. marcescens* ATCC 31453 *ppk1*
SM PPK_rev	atatCTCGAGCTGTCCTGGTTGTTCCAGAG	XhoI	
KP PPK1_fwd	atatCCATGGCAATGGGCCAAGAGAAACTGTATATC	NcoI	*K. pneumoniae* KPNIH1 *ppk1*
KP PPK1_rev	atatCTCGAGATCCGGCTGTTCCAGG	XhoI	
AB PPK1A_fwd	atatCCATGGCAATGACATCATTGCCCAC	NcoI	*A. baumannii* AB5075 *ppk1A*
AB PPK1A_rev	atatCTCGAGCCCAGTTAAGTTCATCAAGTG	XhoI	
AB PPK1B_fwd	atatCATATGATGAATACCGCAATTACCG	BamHI	*A. baumannii* AB5075 *ppk1B*
AB PPK1B_rev	atatGCGGCCGCTTTATTTAAAGGTTTCCAGCAGTTC	EcoRI	
AB PPK2_fwd	atatGGATCCATGAGTGAACAACAACCAAA	BamHI	*A. baumannii* AB5075 *ppk2*
AB PPK2_rev	atatCTCGAGTTAATCTGTCTCTCAGCTGTTT	XhoI	
KP PPK2_fwd	atatGGATCCATGGGCAACAAGAAAAGC	BamHI	*K. pneumoniae* KPNIH1 *ppk2*
KP PPK2_rev	atatCTCGAGTCAAAACTTCTCTGGAATGAAG	XhoI	
T7 chk_fwd	AATACGACTCACTATAGGGG	-	T7 promoter specific
T7 chk_rev	TATGCTAGTTATTGCTCAGC	-	

Oligos – qRT-PCR

Oligo name	Sequence	Efficiency	R^2	Target Gene
qAB 1A_fwd	GGTTATTGAGTTGCGGGCAC	97.9	0.995	*A. baumannii ppk1A*
qAB 1A_rev	TTCACGGCGGACCACTAAAA			
qAB 1B_fwd	CCATACTGCAATTGAGCGCC	104.0	0.995	*A. baumannii ppk1B*
qAB 1B_rev	CGGAAATGGATGGGCTGGAT			
qClpX_fwd (Tipton and Rather 2017)	GCGTTTGAAAGTCGGGCAAT	99.8	0.994	*A. baumannii clpX*
qClpX_rev (Tipton and Rather 2017)	CCATTGCAAACGGCACATCT			

Table S2: Quantification and statistical comparison of bacterial growth curve kinetics. Analysis was performed using AMiGA (Midani, Collins, and Britton 2021).

**Strain comparison**	Growth rates	Sig. Diff. (95.0% CI)
	K. pneumoniae	
**KPNIH1 vs. KPNIH1 + Gallein**	1.51 : 1.33	NO
	***A. baumannii* VIR-O**	
**AB5075 vs. ΔPPK1A**	0.791 : 0.915	NO
**AB5075 vs. ΔPPK1B**	0.788 : 1.07	NO
**AB5075 vs. AB5075 + Gallein**	0.788 : 0.767	NO
**ΔPPK1A vs. ΔPPK1A + Gallein**	1.08 : 0.980	NO
**ΔPPK1B vs. ΔPPK1B + Gallein**	0.915 : 0.976	NO
	***A. baumannii* AV-T**	
**AB5075 vs. ΔPPK1A**	1.55 : 1.60	NO
**AB5075 vs. ΔPPK1B**	1.55 : 1.20	NO
**AB5075 vs. AB5075 + Gallein**	1.56 : 1.80	NO
**ΔPPK1A vs. ΔPPK1A + Gallein**	1.60 : 1.45	NO
**ΔPPK1B vs. ΔPPK1B + Gallein**	1.18 : 1.32	NO

References

Midani, Firas S., James Collins, and Robert A. Britton. 2021. "AMiGA: Software for Automated Analysis of Microbial Growth Assays." *MSystems* 6 (4): e0050821. https://doi.org/10.1128/mSystems.00508-21.

Neville, Nolan, Nathan Roberge, Xiang Ji, Preyesh Stephen, Jiasheng Louis Lu, and Zongchao Jia. 2021. "A Dual-Specificity Inhibitor Targets Polyphosphate Kinase 1 and 2 Enzymes to Attenuate Virulence of Pseudomonas Aeruginosa." *MBio* 12 (3): e0059221. https://doi.org/10.1128/mBio.00592-21.

Robert, Xavier, and Patrice Gouet. 2014. "Deciphering Key Features in Protein Structures with the New ENDscript Server." *Nucleic Acids Research* 42 (Web Server issue): W320-324. https://doi.org/10.1093/nar/gku316.

Tipton, Kyle A., and Philip N. Rather. 2017. "An OmpR-EnvZ Two-Component System Ortholog Regulates Phase Variation, Osmotic Tolerance, Motility, and Virulence in Acinetobacter Baumannii Strain AB5075." *Journal of Bacteriology* 199 (3): e00705-16. https://doi.org/10.1128/JB.00705-16.

Tzeng, C. M., and A. Kornberg. 2000. "The Multiple Activities of Polyphosphate Kinase of Escherichia Coli and Their Subunit Structure Determined by Radiation Target Analysis." *The Journal of Biological Chemistry* 275 (6): 3977–83. https://doi.org/10.1074/jbc.275.6.3977.
